# The Coupled-Resonances Asymmetric Lineshape for Photoemission Spectra

**DOI:** 10.1021/acs.jpcc.5c05560

**Published:** 2025-11-17

**Authors:** Alberto Herrera-Gomez, Dulce Maria Guzman-Bucio, Dagoberto Cabrera-German, Abraham Carmona-Carmona, Vincent Crist, Anthony D. Dutoi

**Affiliations:** † 428213 CINVESTAV-Unidad Queretaro, Libramiento Norponiente 2000, Fracc. Real de Juriquilla, Queretaro 76230, Mexico; ‡ Departamento de Investigación en Polímeros y Materiales, 27813 Universidad de Sonora, Blvd. Luis Encinas y Rosales S/N., Hermosillo, Sonora 83000, Mexico; § 3972 Benemérita Universidad Autónoma de Puebla. Esquina Universal Av San Claudio, Blvrd 22 Sur y, Cd Universitaria, 72592 Heroica Puebla de Zaragoza, Puebla 72000, Mexico; ∥ The XPS Library, Salem, 1091 Vineyard View Way S, Salem, Oregon 97306, United States; ⊥ Department of Chemistry, 7699 University of the Pacific, 3601 Pacific Avenue, Stockton, California 95211, United States

## Abstract

This work introduces the Coupled-Resonances (CR) line shape as a theoretically robust model for analyzing asymmetric peaks in photoelectron spectra, broadly applicable across various materials. The CR line shape extends the conventional Lorentzian distribution by incorporating an interference term that contributes to the peak asymmetry. This new approach addresses limitations of the widely used Doniach- Šunjić (D&S) model, which is often applied beyond its intended scope of metals with high densities of states at the Fermi level due to a lack of viable alternatives. Unlike the DS line shape, the CR model is integrable, enabling its use in precise chemical composition calculations, and it consistently provides superior fits to experimental data. The CR model’s versatility is evident in its ability to simplify to a Lorentzian for a single resonance. However, with multiple resonance states, the total line shape is no longer a simple summation of individual peak contributions. Instead, a significant interference term emerges, profoundly contributing to the observed peak asymmetry and shifting the maximum peak intensity. This highlights the critical need to consider interference terms in multiplet calculations of lineshapes. The CR line shape has been implemented in the freely available software, AAnalyzer. While most asymmetric peaks are accurately described by CR Type-II (two resonances), some require CR Type-III (three resonances) for optimal fitting, as demonstrated in the included examples. Ultimately, the CR model offers a more accurate and versatile approach to analyzing asymmetric lineshapes in photoemission spectroscopy, with broad applicability to a wide range of materials, including metals.

## Graphical abstract

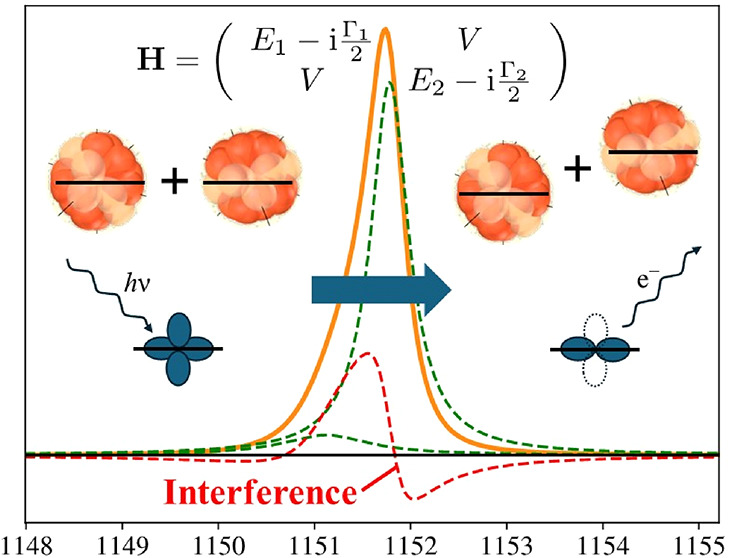

## 1 Introduction

While most peaks in photoelectron spectra are symmetric and accurately described by Voigt profiles, a significant number, particularly those from transition metals, display notable asymmetries. For decades, the Doniach-Šunjić (D&S) line shape has been the primary theoretical model for explaining this phenomenon.^1^ Derived from a simplified version of the Nozières and de Dominicis (N&D) Hamiltonian,^2^ the D&S model attributes asymmetry to many-body interactions in metals, specifically the additional coupling between valence band electrons caused by the sudden creation of a core hole. Through a series of approximations, D&S identified an “infrared catastrophe,” where the energy associated with low-energy transitions diverges, leading to an asymmetry in the photoelectron line shape.^3^

Despite its theoretical appeal, the D&S line shape’s underlying theory is strictly applicable only to metallic systems. Yet, due to a lack of robust theoretical alternatives, it has been widely, and often inappropriately, applied across various types of materials. This widespread use has revealed several practical and theoretical issues, as discussed in detail in reference 3, underscoring the urgent need for a more appropriate theoretical alternative.

The success of the double-Lorentzian (DL) in reproducing experimental asymmetric distributions^3^ prompted us to explore theoretical approaches that could inherently recover the DL line shape. Currently, the field lacks a general theoretical framework capable of describing asymmetric peaks across a wide range of materials—including nonmetallic systems—while simultaneously preserving integrability and allowing for a physical interpretation of fitting parameters. Integrability is particularly crucial for chemical composition analysis, where the nonintegrability of the D&S line shape often leads to poor quantitative accuracy.

A critical aspect often overlooked in line shape calculations from multiplet analyses is the effect of interference between various available states. In a photoemission process, immediately after sudden core-hole creation, the system’s energy is not well-defined; it coexists in multiple final states with different energies. The interference between these states significantly contributes to peak asymmetry. These interference effects are effectively neglected in conventional multiplet calculations, where the total signal is simply summed from each multiplet component.

Bridging this theoretical and practical gap is essential for improving both the precision and reliability of photoelectron spectroscopy. A physically motivated, integrable, and computationally efficient line shape would not only enhance spectral fits but also enable the extraction of meaningful quantities related to materials’ electronic structure. This would deepen our understanding of electron interactions in complex materials and allow for more accurate analysis in systems where peak asymmetry is the rule rather than the exception. In this paper, we propose the Coupled-Resonances (CR) theoretical line shape (Section 2.4). We demonstrate that the CR line shape is not only more theoretically sound but also offers practical advantages: fits require fewer peaks, and—surprisingly—the implementation algorithm is faster (Section 2.5.3).

## 2 Theoretical Framework

### 2.1 Resonances

The relaxation of many types of resonances yields symmetric signals described by the Lorentzian energy distribution. One of the most important appeals of the CR line shape is that it is a natural asymmetric extension of the Lorentzian, which is the line shape typically associated with lifetime broadening. Labeling states with finite lifetimes as resonances originally refers to scattering theory, in which a material particle or the energy of a photon is briefly trapped before moving on due to a (near) matching of frequencies/energies. At a high level of abstraction, all problems are scattering or resonance problems; molecular excitations are simply the temporary capture of resonant photons, and ionization couples the capture of a photon from the electromagnetic continuum to the release of an electron into its free-particle continuum. There is no requirement that the incoming particles be of the same number or identity as the outgoing ones. One speaks of decay channels to distinguish the different ways that a resonance can decay.

XPS concerns a resonance where an incoming photon results in the release of a (multiply scattered) primary photoelectron, plus subsequent electrons and/or photons associated with the decay of a remaining core-hole state. This “initial” core-hole is the resonance state that we are concerned with, since its properties determine the fundamental line shape of the primary photoelectron by conservation of energy. The core-hole state of the remaining solid is coupled to the continuum through various channels (i.e., Auger and radiative decays), and it does not persist indefinitely.^4^ Having a finite lifetime, one would naively expect to associate it with a Lorentzian energy distribution. However, as we will demonstrate, similar decaying states, perhaps distinguished by different groups of multiplets of valence *p*, *d*, or *f* electrons on the core-hole site, may be coupled with each other. At extremely short time scales, the quantum system does not settle into a single, well-defined energy state. Instead, it oscillates rapidly among various possible final configurations, each with a different energy. This occurs because the system’s wave function exists as a superposition of these states before it collapses into a specific eigenstate of the Hamiltonian. This would mean that different channels (having different final states of the material) are coupled on the same time scale as the decay process, and this can naturally lead to interference effects and a broader library of possible photoelectron lineshapes. We have developed a formalism that precisely accounts for the impact of this rapid back-and-forth movement between states on the observed line shape.

The theory of resonances is large and mature,^5,6^ and the fundamentals are described in standard quantum mechanics textbooks, both phenomenologically^7^ and formally.^8^ This article applies some of the most basic features, leaving space for the aspects of this intricate field that are not yet fully understood in our specific context. Most broadly, resonance states are characterized by a complex energy. The practical implications are worked through below, but some comments on the theory are in order. Complex energies arise when a choice is made to handle a problem with a coupling to a true continuum by focusing explicit attention only on a subspace of states that represent normalizable, “quasi-bound” (i.e., resonance) states. Though complex energies are often thought of as an unphysical artifact of the down-projection from a larger problem, strictly speaking, this is not itself an approximation; Hermiticity of operators can only be asserted in the space of square-integrable functions that do not include true continua. As such, appropriately handled, these energies have a clear interpretation, which we attempt to clarify in a language as close as possible to the more familiar Hermitian form of quantum mechanics.

### 2.2 The Energy Distribution in Terms of the Autocorrelation Function

This section describes the relationship between the autocorrelation function and the energy distribution function of the initial core-hole resonance state of the solid |ϕ­(*t* = 0)⟩ (assumed normalized) (Figure 1).

**Figure 1:**
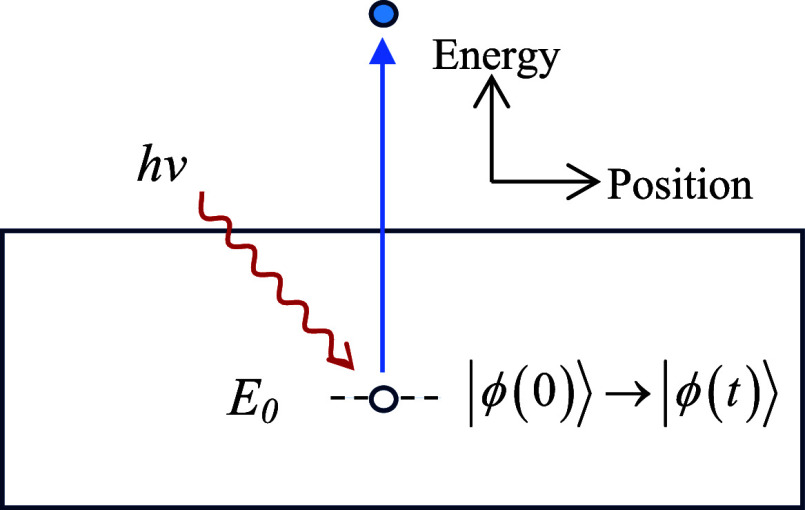
Diagrammatic representation of the state of the solid after photoelectron emission.

Since the state decays in time, it is not an eigenstate of the Hamiltonian. Therefore, it can be expanded in terms of the familiar real-energy eigenstates of the Hamiltonian; these states are members of a continuum (*E* > 0), whose asymptotic boundary conditions describe plane-wave states of incoming and outgoing particles. Though any state of energy *E* is highly degenerate, some unique linear combination of them will project onto |ϕ(0)⟩, and this is defined as the delta-normalized state |*E*⟩, so that we can write

$$\left|\;\phi \left(0\right)\right\rangle =\int_{0}^{\infty }\mathrm{dE}\;f\left(E\right)\left|E\right\rangle ,\mathrm{with}\left\langle E\right|E’\rangle =\delta \left(E-E’\right)$$

The energy distribution of the core-hole resonance state is |*f*(*E*)|^2^, and this plays a fundamental role since, as described in Section 2.3.2, it defines the energy distribution of the photoelectron ejected from this core hole. For convenience in recognizing the Fourier transformation later, we can extend the lower integration bound to –∞ without practical consequence, since the function *f*(*E*) is presumed to be tightly peaked at an energy well above zero. The state evolves with time as follows:

$$\left|\phi \left(t\right)\right\rangle =\int_{-\infty }^{\infty }\mathrm{dE}e^{-iEt/\hbar }f\left(E\right)\left|E\right\rangle$$

We can therefore write |*f* (*E*)|^2^ in terms of the autocorrelation function ⟨ϕ(0)|ϕ­(*t*)⟩ as follows

$$\left\langle \phi \left(0\right)\right|\phi \left(t\right)\rangle =\left[\int_{-\infty }^{\infty }\mathrm{dE}’f^{*}\left(E’\right)\left\langle E’\right|\right]\left[\int_{-\infty }^{\infty }\mathrm{dE}e^{-i\mathrm{Et}/\hbar }f\left(E\right)\left|E\right\rangle \right]$$

yielding

1 $$\left\langle \phi \left(0\right)\right|\phi \left(t\right)\rangle =\int_{-\infty }^{\infty }\mathrm{dE}{\left|f\left(E\right)\right|}^{2}e^{-i\mathrm{Et}/\hbar }$$

With a change of variable ν = *E*/*h*, we immediately recognize the right-hand side as the definition of the Fourier transform of |*f*(*E*)|^2^, which we can invert to obtain:

$${\left|f\left(E\right)\right|}^{2}=\frac{1}{2\pi \hbar }\int_{-\infty }^{\infty }\mathrm{dt}\;e^{i\mathrm{Et}/\hbar }\left\langle \phi \left(0\right)\right|\phi \left(t\right)\rangle$$ 2

### 2.3 The Lorentzian as the Fundamental Line Shape for Symmetric Peaks

The relationship between the time and energy domains that provided the above energy distribution function in terms of the autocorrelation function is general for any state built of continuum eigenstates, not requiring any special features of non-Hermitian quantum mechanics. This section provides a detailed exposition of the theoretical framework underlying the Lorentzian line shape, which is the fundamental symmetric line shape in photoelectron and other spectroscopies and is characterized by a complex energy. This will lay the groundwork for the derivation of the Coupled-Resonances (CR) line shape presented in Section 2.4.

#### 2.3.1 The Lorentzian Energy Distribution

A Lorentzian energy distribution can be derived from the following assumptions about the autocorrelation function:

- The relevant resonance *state* |ϕ(0)⟩ can be described in terms of orbital occupancy or vacancy, which are independent of the physical *process* in a given experiment. (The spectral signature indicating that such a state has been accessed—photon absorption, electron emission, etc.—can, of course, depend on these details.) This implies that this state has no inherent notion of forward or backward time (no net momenta), and time-symmetric behavior of all observables is then expected.
- The probability that the state at time *t* remains in its initial state, i.e., |⟨ϕ(0)|ϕ­(*t*)⟩|^2^, decays exponentially with time because the linearity of the Schrödinger equation dictates that the rate of population transfer is proportional to the population of the state itself. Population that is transferred to a continuum is assumed to never return.
- The average energy *E*_0_ for the state defines a frequency *E*_0_/*h* that describes the phase oscillation of the state.

The autocorrelation function that satisfies the above conditions can be expressed in the following way:

$$\left\langle \phi_{I}\left(0\right)\right|\phi_{I}\left(t\right)\rangle =e^{-iE_{0}t/\hbar -\Gamma |t|/2\hbar }\left(\Rightarrow {\left|\left\langle \phi_{I}\left(0\right)\right|\phi_{I}\left(t\right)\rangle \right|}^{2}=e^{-\Gamma |t|/\hbar }\right)$$ 3

where the subscript *I* denotes that only a single resonance is being considered in this case. This oscillates with frequency *E*_0_/ℏ and decays exponentially in both time directions with characteristic resonance lifetime *h*/Γ (Figure 2). Its maximum value is 1 at *t* = 0, as it must be; however, the discontinuity in its first derivative is an unrealizable idealization (akin to a δ-function position state being a useful fiction). Keeping in mind that Fourier transforms invert their coordinate systems (making narrow features broad and vice versa), this cusp at *t* = 0 is directly responsible for the far off-resonant tails of the resulting energy distribution. If the cusp were to be smoothed, it would dampen these tails, and this damping would occur more quickly if the smoothing of the cusp were broader. Experience observing actual Lorentzians therefore shows that, to within measurement noise of the tails of the line shape, true exponential decay takes over already at commensurately short times.

**Figure 2:**
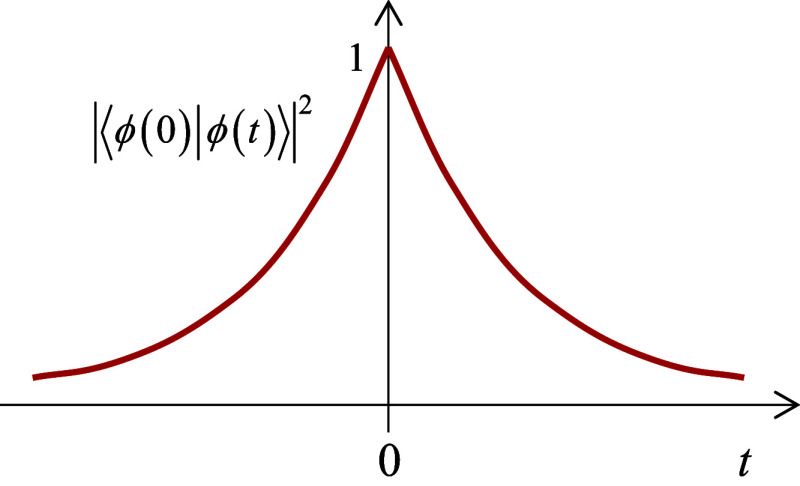
Autocorrelation function of the state of the solid decays exponentially in both directions of time.

Inserting the assumed autocorrelation function (eq 3) into eq 2 now yields the familiar Lorentzian function

4 $${\left|f_{I}\left(E\right)\right|}^{2}=\frac{1}{\pi }\frac{\frac{\Gamma }{2}}{{\left(E-E_{0}\right)}^{2}+{\left(\frac{\Gamma }{2}\right)}^{2}}$$

The integral of this function over all energies is 1, in agreement with its interpretation as the energy representation of a normalized state. Although our starting assumptions seem rather stringent, it should be noted that spectral lineshapes directly describe their associated autocorrelation functions via eq 1. A Lorentzian line shape is therefore direct evidence of exponential decay in both directions in time (albeit hypothetical for the reverse direction), and the peak position is indicative of phase oscillations. This then strictly validates our assumptions above, at least as a physically meaningful idealization.

#### 2.3.2 Energy of the Photoelectron

Once the energy distribution of the core-hole state has been assessed, it is necessary to invoke entanglement to obtain the energy distribution of the photoelectron. For a given photon energy *hv*, the probability that the electron ends up with an energy *K’* in the *K*–*dK* < *K*′<*K* range is equal to the probability that the solid ends up with energy *E’* in the *E* < *E*′<*E* + *dE* range; i.e., the photoelectron line shape |*f*_elec_(*K*)|^2^ also has a Lorentzian form, centered at *K*_0_ = *hv*–*E*_0_.

### 2.4 The Construction of the Coupled-Resonances Line Shape

#### 2.4.1 From Single to Multiple Resonances

Inspired by the successful use of a double-Lorentzian line shape to reproduce asymmetric peaks,^3^ we, a theorist and a team of experimentalists, sought a firm theoretical foundation for this empirical approach. Our initial exploration involved eq 1 to obtain the autocorrelation function associated with the DL energy distribution described in eq 2 of ref 3. This has an asymptotic *t* ^–3^ decay, due to the discontinuity in the second derivative at the peak maximum, which is physically inconsistent with the paradigm of exponentially decaying population for a resonance state. Removing the derivative discontinuities of the DL with a smooth switching function resolved the *t* ^–3^ issue and thereby provided some insight into the structure of the interferences in the time domain (specifically, there must be an antisymmetric imaginary component to the autocorrelation function). However, this did not provide a direct underlying physical model. With the understanding that we were seeking an interference effect, single-particle scattering was investigated in the energy domain for a variety of one-dimensional potentials designed to have multiple decay exponents. This yielded only symmetric Lorentzian energy distributions, which are consistent with symmetric time behavior (the Fourier transform of a real symmetric function is real and symmetric). Given that the target problem involves a large number of electrons in three dimensions, the breakthrough was to postulate the existence of two (or more) resonance states close enough in energy that, if coupled, their decays could interfere in time. This yielded a robust and successful theoretical framework for asymmetric lineshapes. This coupled-resonance line shape has since been implemented in a practical peak-fitting software^9^ allowing us to test it on real data and confirm its ability to accurately reproduce experimental spectra.

In the theory of isolated resonances, as discussed so far, a single Lorentzian peak as given in eq 3 would be generated by the time dynamics from the 1 × 1 matrix Hamiltonian for one resonance

5 $$H=\{\begin{array}{ll} H_{+}=\left(E+i\frac{\Gamma }{2}\right) & t<0\\ H_{-}=\left(E-i\frac{\Gamma }{2}\right) & 0\leq t \end{array}$$

The minus sign corresponds to positive times, and the plus sign to negative times (accounted for by taking the absolute value of *t* in eq 3). The Hamiltonian for the full system can be thought of as a very large (infinite) matrix that couples the initial state to a continuum of other states. The inclusion of Γ in a finite discrete representation represents a quasi-bound state, allowing for a very simplified and powerful treatment of the decay of the population of this state to the implicit continuum.

The Hamiltonian in eq 5 can be naturally generalized to *N* resonances:

$$\varphi_{1}=\left(\begin{array}{l} 1\\ 0\\ 0\\ ...\\ 0 \end{array}\right),\varphi_{2}=\left(\begin{array}{l} 0\\ 1\\ 0\\ ...\\ 0 \end{array}\right),...,\varphi_{N}=\left(\begin{array}{l} 0\\ 0\\ 0\\ ...\\ N \end{array}\right)$$ 6

as follows:

7 $$H_{\pm }=E\pm \frac{i}{2}\Gamma =\left(\begin{array}{lllll} E_{1} & V_{12} & V_{13} & ... & V_{1N}\\ V_{12}^{*} & E_{2} & V_{23} & ... & V_{2N}\\ V_{13}^{*} & V_{23}^{*} & E_{3} & ... & V_{3N}\\ ... & ... & ... & ... & ...\\ V_{1N}^{*} & V_{2N}^{*} & V_{3N}^{*} & ... & E_{N} \end{array}\right)\pm \frac{i}{2}\left(\begin{array}{lllll} \Gamma_{1} & 0 & 0 & ... & 0\\ 0 & \Gamma_{2} & 0 & ... & 0\\ 0 & 0 & \Gamma_{3} & ... & 0\\ ... & ... & ... & ... & 0\\ 0 & 0 & 0 & 0 & \Gamma_{N} \end{array}\right)$$

in which **E** is Hermitian and **Γ** is diagonal. The discrete normalizable states (eq 6) are coupled to each other through the *V*_*ij*_ parameters. By adequately choosing the phase of the states, we can choose this off-diagonal part of **E** to be real without loss of generality. The {Γ_*j*_} accounts for the decays of these resonance states, each ostensibly into its own independent implicit continuum (discussed immediately below). We chose **φ**_1_ (eq 6) as the initial state. It can be any of those listed in eq 6, but a combination of them is outside of our current model because it is identical to choosing a nondiagonal **Γ** (via a unitary transformation).

The eigenstates of the reverse-time (*t* < 0) Hamiltonian in eq 7 are represented as

8 $$\left\{\Phi_{1},\Phi_{2},\Phi_{3},\Phi_{4},...\right\}$$

and the corresponding eigenvalues as {ε_1_,ε_2_,ε_3_,ε_4_,···} (the eigenvectors and eigenvalues of the forward-time Hamiltonian are their complex conjugates). *E*_*R*_*j*__= Re­(ε_*j*_) is the energy of the eigenstate **Φ**_*j*_ and Γ_*Rj*_ ≡ 2 Im (ε_*j*_) is proportional to its decay rate (note that a sign convention here depends on the fact that we have specified the reverse-time Hamiltonian). Since the states correspond to multiplet configurations of the core hole-valence band system, the energies {*E*_*R*_*j*__} can be identified as the central energy of multiplet groups. As discussed in Section 2.5.2, the widening caused by the energy spread of these groups is reflected during peak-fitting in a larger value of the {Γ_*R*_*j*__}.

The Hamiltonian in eq 7 can be classified depending on the number of resonances. In this way, CR type I corresponds to the Lorentzian line shape. The asymmetries found in the spectra of elements in the periodic table’s fourth, fifth, and sixth rows, including metal and metal oxides, can be reproduced using mainly CR type II but sometimes CR type III is required.

#### 2.4.2 Derivation of the CR Line Shape

For the case of *N* resonances, the initial state **φ** = (1,0,···,0) can be written as a linear combination of the eigenstates {**Φ**_1_,**Φ**_2_,···,**Φ**_*N*_} of the Hamiltonian as follows

9 $$\left|\phi \left(0\right)\right\rangle \Leftrightarrow \varphi \left(0\right)=\left(\begin{array}{l} 1\\ 0\\ ...\\ 0 \end{array}\right)=\sum_{j=1}^{N}\alpha_{j}\Phi_{j}$$

By applying the time-evolution operator, we get:

$$\left|\phi \left(t\right)\right\rangle =e^{-iHt/\hbar }\left|\phi \left(0\right)\right\rangle \Leftrightarrow \varphi \left(t\right)=\overset{N}{\underset{j}{\sum }}\alpha_{j}e^{-i\varepsilon_{j}t/\hbar }\Phi_{j}$$ 10

To assess the line shape, it is necessary to calculate the autocorrelation function, which is done as follows:

$$\left\langle \phi \left(0\right)\right|\phi \left(t\right)\rangle =\varphi^{†}\left(0\right)\cdot \varphi \left(t\right)={\left(\overset{N}{\underset{j}{\sum }}\alpha_{j}\Phi_{j}\right)}^{†}\cdot \overset{N}{\underset{m}{\sum }}\alpha_{m}e^{-i\varepsilon_{m}t/\hbar }\Phi_{m}$$ 11

In eqs 10 and 11, it is implied that the eigenvalues and eigenvectors corresponding to the appropriate sign of *t* are used, which can be shown to provide for ⟨ϕ(0)|ϕ­(−*t*) ⟩= ⟨ϕ(0)|ϕ­(*t*)⟩*. It is also important to realize that the eigenvectors of the non-Hermitian Hamiltonian are not orthogonal to each other, and this underlies the asymmetry of the CR lineshapes. Therefore, the cross terms in eq 11 should be kept.

By expanding the product in eq 11 we get the following:

$$\left\langle \phi \left(0\right)\right|\phi \left(t\right)\rangle =\overset{N}{\underset{m=1}{\sum }}e^{-i\varepsilon_{m}t/\hbar }\left(\overset{N}{\underset{j=1}{\sum }}\alpha_{j}^{*}\alpha_{m}\Phi_{j}^{†}\Phi_{m}\right)=\overset{N}{\underset{m=1}{\sum }}e^{-i\varepsilon_{m}t/\hbar }X_{m}$$ 12

where the complex intensities {*X*_*m*_} are defined as follows:

13 $$X_{m}\equiv \sum_{j=1}^{N}\alpha_{j}^{*}\alpha_{m}\Phi_{j}^{†}\Phi_{m}=\varphi^{†}\left(0\right)\Phi_{m}\alpha_{m}$$

It can immediately be recognized from eq 12 that the {*X*_*m*_} must sum to 1 to obey the boundary condition on the autocorrelation at *t* = 0 (this can be derived explicitly for a normalized initial state because left and right diagonalizing transformations are mutually inverse). The CR line shape can now be found by using eq 12 in eq 2

$${\left|f_{N}\left(E\right)\right|}^{2}=\int_{-\infty }^{\infty }\mathrm{dt}\;e^{iEt/\hbar }\overset{N}{\underset{m=1}{\sum }}e^{-i\varepsilon_{m}t/\hbar }X_{m}$$ 14

where the subindex *N* stands for the CR type *N* line shape. By carrying out the time integrals, we reach the following expression

15 |fN(E)|2=1π∑m=1N[Re(Xm)ΓRm2(E−ERm)2+(ΓRm2)2+Im(Xm)(E−ERm)(E−ERm)2+(ΓRm2)2]

(The derivation of this equation is provided in the Supporting Information.) In all cases, the CR distribution integrates to 1, since the sum of Re­(*X*_*m*_) is 1, and each interference term individually integrates to zero. Finally, the condition that the sum of Im­(*X*_*m*_) is 0 provides for the far-off resonant tails of this distribution decaying as *E* ^–2^ (as for single Lorentzians), even though the individual interference terms decay as *E* ^–1^. (We note a sign convention: if the forward-time Hamiltonian is used to define the eigenstates, the X_*m*_ are taken to their conjugates, and there is a negative sign preceding Im­(X_*m*_) in this equation.) The total line shape consists of a series of (symmetric) Lorentzian peaks plus antisymmetric components centered at each resonance energy. In the limit of no off-diagonal couplings, when the initial state is an eigenstate, this collapses to a single Lorentzian.

#### 2.4.3 CR Type II Example

For CR Type II, the Hamiltonian of eq 7 has the following form

16 $$H_{\pm }=\left(\begin{array}{ll} E_{1}\pm i\frac{\Gamma_{1}}{2} & V_{12}\\ V_{12} & E_{2}\pm i\frac{\Gamma_{2}}{2} \end{array}\right)$$

Peak-fitting requires finding the parameters {*E*_1_, *E*_2_, Γ_1_, Γ_2_, *V*_12_} that yield the eigenvectors {**Φ**_1_,**Φ**_2_} and eigenvalues {ε_1_,ε_2_} that reproduce the experimental line shape. This is done through an optimization process described in Section 2.5.3. For a given set of the {*E*_1_, *E*_2_, Γ_1_, Γ_2_, *V*_12_} parameters, the eigenvectors and eigenvalues of the Hamiltonian are found, as well as the parameters {α_1_,α_2_} of eq 9. As described by eq 14, the knowledge of {**Φ**_1_,**Φ**_2_}, {ε_1_,ε_2_}, and {α_1_,α_2_}, allows for the assessment of a line shape. This line shape is compared with the experimental data and, through an optimization process, the appropriate set of parameters {*E*_1_, *E*_2_, Γ_1_, Γ_2_, *V*_12_} are found. The optimized values of the parameters {*E*_1_, *E*_2_, Γ_1_, Γ_2_, *V*_12_} yield the corresponding final sets {**Φ**_1_,**Φ**_2_}, {ε_1_,ε_2_}, and {X_1_,X_2_}. The real part of the eigenvalues corresponds to the energy of the solid final states.

An example decomposition is shown in Figure 3 for the following parameters *E*_1_ = 1151.6 eV, *E*_2_ = 1151.3 eV, *V*_12_ = 0.43 eV, Γ_1_ = 0.1 eV, and Γ_2_ = 1.5 eV. The eigenvalues of the (reverse-time) Hamiltonian are ε_1_ = (1151.78 + 0.24*i*)­eV and ε_2_ = (1151.12 + 0.56*i*)­eV; in this way, the energies of the two configurations of the core hole in eq 9 are *E*_*R*_1__ = 1151.78 eV and *E*_*R*_2__ = 1151.12 eV, and the corresponding lifetime broadenings are Γ_*R*_1__/2 = 0.24 eV and Γ_*R*_2__/2 = 0.56 eV. It is the lifetime broadening of the resonances that should be compared with ab initio calculations; however, there are no available calculations for asymmetric peaks.^10^ Note that the maximum of the CR line shape does not align with the energy of the resonance, but is shifted by the antisymmetric terms.

**Figure 3:**
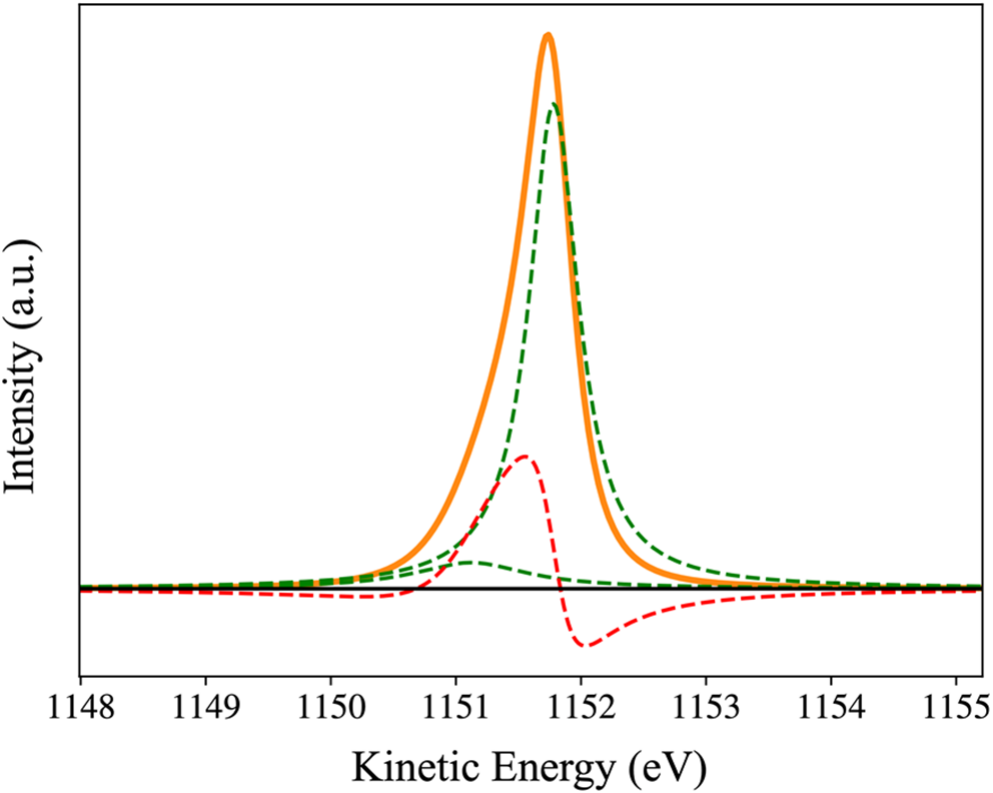
Overall peak shape for the parameters in the text is shown as a solid orange line. The two Lorentzian contributions from eq 15 are shown as dashed green lines, and the combined interference terms are shown as a single dashed red line. The asymmetry of the peak is dominated by the antisymmetric contributions.

As discussed in the Supporting Information, in some cases, the Γ_1_ or Γ_2_ obtained from fitting experimental lineshapes are negative, but always lead to positive (correct) signs of Γ_*R*_1__ and Γ_*R*_1__. This means that *any* state described by such a Hamiltonian will decay in time, but it is not presently understood how to interpret the meanings of the negative Γ_1_ or Γ_2_ in the original Hamiltonian. This is an area of present study, and the leading conjectures are that (1) there is no conflict because only the eigenstates must unconditionally decay in time, and, in fact, the decay parameters in the Hamiltonian parameters do not *individually* have straightforward interpretation; (2) the negative decay parameters are functioning as a proxy for some mechanism that would have a straightforward interpretation in a model that included more states; or (3) the unused parameters in the initial state vector (equivalently, nondiagonal **Γ**) could be used to fit these peaks. The latter of these three conjectures has been preliminarily explored with the result that there is no exact transformation from the negative-Γ fits obtained in our experimental work to a more general model with only positive Γ values. It remains to be seen if numerically acceptable results can be found. However, by expanding the model, the risk of overfitting the data arises, which then limits how much information we can convincingly extract about the underlying Hamiltonian.

#### 2.4.4 The Antisymmetric Terms

The antisymmetric terms in eq 15, not the additional Lorentzians, are the dominant contribution to the peak asymmetry. They are proportional to the imaginary part of the complex intensities {*X*_*m*_}. From eq 13, it follows that this imaginary part, and therefore the asymmetry in photoelectron peaks, originates from the lack of orthogonality of the eigenvectors

17 Im(Xm)=Im(αm*αmΦm†Φm+∑j≠mNαj*αmΦj†Φm)=Im(∑j≠mNαj*αmΦj†Φm)≠0

Besides “antisymmetric terms” (because of eq 15), we also indistinctively call them “cross terms” (because of eq 17) and “interference terms”; the latter is because the state simultaneously coexists in various resonances (eq 9).

In Hermitian quantum mechanics, the eigenstates of the Hamiltonian are inherently orthogonal. However, this orthogonality breaks down in the context of the non-Hermitian Hamiltonian described in eq 7. This nonorthogonality is a direct consequence of the imaginary energy terms present in eq 7. These imaginary terms are introduced to account for the continuum states that are excluded from the explicit formulation of the Hamiltonian.

If a complete set of states *N*_∞_, including the continuum (i.e., with *N*_∞_ approaches infinity), were incorporated into the Hamiltonian, the imaginary energy component would be unnecessary, and the eigenstates would retain their orthogonality. Non-Hermitian quantum mechanics attempts to provide an approximate solution by considering only the first *N* eigenstates of the complete Hamiltonian and accounting for the rest of the *N*_∞_ states by using complex energies (so the Hamiltonian reduces to that in eq 7). The observed lack of orthogonality can be heuristically understood by considering the implications for the dot product (or inner product) between eigenstates. When we truncate the system to only the *N* resonances specified in eq 7, we effectively neglect the contributions from the continuum states to this dot product.

Since the dot product between two eigenstates of the Hamiltonian in eq 7 is not zero, it implies that the contributions from the continuum states to the full, complete-space dot product also cannot be zero. This suggests an overlap between the continuum states to which each resonance is coupled. Physically, this can be interpreted as the eigenstates of the Hamiltonian in eq 7 being interconnected through the continuum of states to which they are individually coupled.

### 2.5 Extrinsic and Intrinsic Broadening of the CR Line Shape

#### 2.5.1 Extrinsic Broadening

To accurately model experimental peaks, the CR line shape must be convolved with a Gaussian distribution to account for extrinsic broadening effects such as X-ray dispersion and spectrometer resolution.^11^ Therefore, the CR line shape must be convolved with a Gaussian distribution whose width is assessed through peak-fitting; this width corresponds to the square root of the sum of the squares of the widths of the various broadening sources. For simplicity, the term ″CR line shape″ will henceforth also refer to the CR line shape after convolution with a Gaussian distribution. This contrasts with the distinct naming convention used for Lorentzian and Voigt distributions.

#### 2.5.2 Broadening Caused by the Energy Distribution of the Multiplet States

The energy distribution of the multiplet states is a source of intrinsic broadening. Each resonance **Φ**_*i*_ corresponds to a multiplet state belonging to a multiplet family; these families are centered at a particular central energy. Since it is not possible to resolve the individual multiplet states, peak-fitting can only assess this central energy; the energy distribution of the multiplet family is reflected in an increased value of the corresponding Γ_*R*_*j*__; however, the Gaussian obtained through peak-fitting accounts for part of this broadening.

#### 2.5.3 Computational Implementation of the Broadening

Generating CR lineshapes for peak-fitting analysis is computationally efficient, requiring one fewer Fast Fourier Transform (FFT) operation than traditional convolution methods. This efficiency arises from the Convolution Theorem, which applies to both forward and inverse Fourier transforms. While convolution can be performed using Fourier transforms of both the CR and Gaussian lineshapes, eq 1 offers a more efficient approach. By leveraging the fact that the autocorrelation function is the inverse Fourier transform of the CR line shape (eq 1), we can directly multiply it by the inverse Fourier transform of the Gaussian function. Applying a forward Fourier transform to this product yields the desired Gaussian-broadened CR line shape, streamlining the process by eliminating one FFT operation. For this reason, it is more efficient to generate the CR line shape using eq 1 than using eq 15.

CR types II and III have been implemented in the AAnalyzer software^9^ available for free at https://xpsoasis.org/download. The CR parameters are optimized alongside other peak-envelope parameters—including peak intensities, energies, parameters defining the line-shape of additional peaks, and those defining the background—to better reproduce the spectrum. The optimization is done using least-squares fitting, minimizing the χ^2^ function of merit

18 $$\chi^{2}=\sum_{i=1}^{N}\left(\frac{S_{i}-S\left(E_{i};a_{1},...,a_{M}\right)}{\sigma_{i}}\right)$$

Where *S*_*i*_ is the total count of the spectrum at energy *E*_*i*_, σ_*i*_ is the intrinsic standard deviation (assuming Poisson statistics), and *S* is the expected intensity at energy *E*_*i*_ assuming that the true parameters are {*a*_*i*_,.···, *a*_*M*_}. The Levenberg–Marquardt Method is employed to find the parameter set that minimizes χ^2^. In addition to fitting parameters and the resulting Hamiltonian eigenvalues, AAnalyzer provides their uncertainties using the covariance matrix method.^12^

As discussed in Section 4, most asymmetric peaks in metal spectra required CR type II lineshapes, while some required type III. The fits presented in this section can be downloaded from the associated discussion forum at https://xpsoasis.org/.

### 2.6 Difference between Systems Leading to Symmetric and Asymmetric Lineshapes

As shown in Section 2.4.2, if the initial |ϕ(0)⟩ state is a linear combination of eigenstates of the Hamiltonian, as in eq 9, the resulting line shape is asymmetric. However, if, for example, the initial state is **Φ**_1_ (i.e., α_1_ = 1 and α_2_ = 0 in eq 9), the system remains in that state oscillating with frequency Re­(ε_1_)/ℏ with a decaying rate of 2Im­(ε_1_)/ℏ, which is precisely the case discussed in Section 2.3 that leads to symmetric Lorentzian lineshapes. Therefore, the difference between the systems leading to symmetric and asymmetric lineshapes is whether the initial state is or is not an eigenstate of the Hamiltonian. Therefore, a condition for the existence of asymmetric lineshapes is that there are distinct resonance states with different central energies.

Interfering core-hole resonances with distinct energies can arise due to interactions with valence electrons (for example), if the state of the combined core-hole–valence system produced by the X-ray perturbation is not an eigenstate. The core-hole potential can break the degeneracy between configurations of valence electrons, or more generally simply change the level structure. In a time-dependent picture, during the time that the core hole decays by (Auger or photon emission, etc.), one can imagine the valence electrons oscillating between multiple basis states, coupled by the presence of the transient core hole.

This is diagrammatically described in Figure 4 for the simplest case: a valence band with two states and one electron. In general, there may be more than one state of multiple valence electrons at play. The key factors are (1) multiple arrangements of valence electrons must be possible, and (2) the core hole must be asymmetric to affect those different arrangements differently.

**Figure 4:**
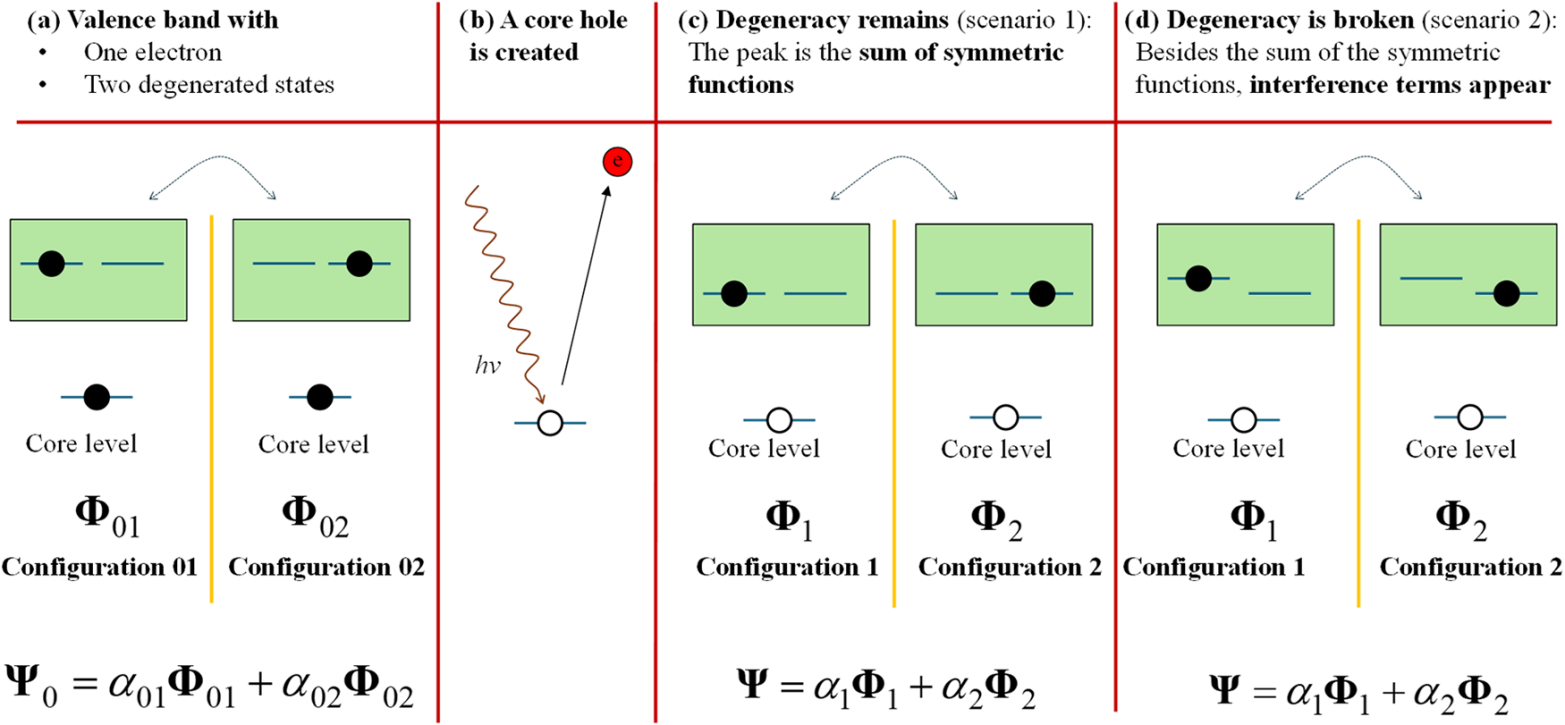
A system consisting of one core level and a valence band with two states and one electron is used to exemplify cases in which interference terms contributing to peak asymmetry appear. (a) The initial system hops between the two possible degenerate configurations. (b) A core hole is created through photoemission. (c) In Scenario I, the presence of the core hole does not break the degeneracy. Although the system hops between the two configurations, no interference terms appear in the line shape. This is typical of *s*-type core holes because spherical symmetric wave functions affect different valence band levels in approximately the same way. (d) In Scenario II, the presence of the core hole breaks the degeneracy, causing interference terms to appear in the peak line shape. This is typical of *p*, *d*, and *f*-type core levels in open *p*, *d*, and *f*-type valence bands because localized wave functions interact differently depending on the specific symmetry of the valence band electron wave functions.

The presence of the core hole not breaking degeneracy, i.e., Figure 4b, is expected in the following cases:

- Core holes from *s*-type orbitals. Due to their spherical symmetry, s-orbital core holes uniformly lower the energy of all valence band electrons, without breaking degeneracy. Graphene is a notable exception; although the core-hole originates from a 1s orbital, the degenerate states forming its π-electronic structure are spatially and energetically susceptible to perturbations. In such cases, the presence of a core hole can affect these degenerate states unevenly, effectively breaking the symmetry and leading to asymmetric lineshapes.
- Systems with a fully occupied valence band. Since the valence electrons completely fill the available states, the system can exist only in a single configuration with a well-defined energy. As a result, no degeneracy is present that could be lifted by the core hole, and the line shape remains symmetric.

Figure 4c illustrates a scenario where the interaction between the core hole and valence band electrons varies, lifting the degeneracy of the valence band states. This differentiated electrostatic interaction arises when asymmetric core holes are localized (e.g., from *p*, *d*, or *f* orbitals) and interact with unfilled valence bands with quantum levels of *p*, *d*, or *f* symmetry. Consequently, asymmetric lineshapes are expected to be observed in systems exhibiting multiplet satellites, which is experimentally the case.

### 2.7 Relation with the Doniach-Šunjić Approach

For metals, these resonances might correspond to excitations near the Fermi level. Under the notation employed by D&S, the Hamiltonian proposed by N&D to describe the coupling among valence band states is the following:^2^

19 $$H_{\mathrm{N\&D}}=\sum_{p}\varepsilon_{p}c_{p}^{†}c_{p}+E_{b}b^{†}b+\sum_{p,p’}V_{p,p’}b^{†}bc_{p}^{†}c_{p’}$$

where *b* ^†^ is the hole creation operator, {c_p_^†^} are the valence band electron creation operators, *E*_*b*_ is the core-hole energy, and {ε_p_} are the energies of the valence band electrons. The first two terms correspond to the energy of the particles when coupling is neglected. The last term describes the matrix element of the transition of a valence band electron from state **p′** to **p** in the presence of a core hole. D&S approximated the ***V***_**p,p′**_ matrix elements as constant, resulting in the following Hamiltonian:^1^

20 $$H_{\mathrm{D\&S}}=\sum_{p}\varepsilon_{p}c_{p}^{†}c_{p}+E_{b}b^{†}b+\frac{v}{N}\sum_{p,p’}b^{†}bc_{p}^{†}c_{p’}$$

where ν is a constant related to the coupling strength and *N* is the number of electrons in the valence band. Through a series of approximations, they obtained the following photoelectron cross-section

21 $$\frac{d\sigma }{d\Omega }∼\{\begin{array}{ll} \frac{1}{{\left(K_{0}-K\right)}^{1-\alpha }} & K<K_{0}\\ 0 & K_{0}\leq K \end{array},\mathrm{with}\;0<\alpha <1$$

where α is a parameter that depends on the electronic structure of the metal. This is the source of the D&S approach’s asymmetry. A significant issue with this cross-section is that the integral under this curve is infinite for any value within the allowed range for α. The final line shape proposed by D&S (eq 18 in ref 1.) inherits this lack of integrability, which makes it unsuitable for chemical composition calculations.

To justify convolving eq 21 with a Lorentzian, D&S stated that *“··· In practice both types of event will be rounded out by lifetime effects···”*. As discussed in Section 3.2, this requires that each of the states considered in eq 20 (∼*N* ^2^) is coupled to a continuum of states, i.e., each one corresponds to a resonance in eq 7 with a total of ∼*N* ^2^ resonances. The comparison with the CR approach can be done through a series of associations between the states considered in eq 19 and eq 7 as described in Table 1. Under the N&D approach (eq 19), the core-hole state is coupled with each excited state of the valence band; however, it does not consider coupling among the excited states of the valence band. In contrast, under the CR approach each resonance in eq 7 is coupled with each other. For this reason, the N&D approach lacks terms in the last two columns of Table 1.

**Table 1:** Association of the Core-Hole States of the Solid Considered in the N&D Framework (eq 19) with CR States (eq 7)­^a^

		state	energy	lifetime width	coupling with state 1	coupling with state 2	coupling with state 3
1	N&D	core-hole	*E*_*b*_	γ/2			
	CR	resonance configuration 1	*E*_1_	Γ_1_			
2	N&D	core-hole plus *p*′_1_→*p*_1_	*E_b_* + (ε_*p*_1__–ε_*p*′_1__)	γ/2	*V*_**p**_1_,**p**′_1__		
	CR	resonance configuration 2	*E*_2_	Γ_2_	*V*_12_		
3	N&D	core-hole plus *p*′_2_→*p*_2_	*E_b_* + (ε_*p*_2__–ε_*p*′_1__)	γ/2	*V*_**p**_1_,**p**′_2__		
	CR	resonance configuration 3	*E*_3_	Γ_3_	*V*_13_	*V*_23_	
...	...	...	...	...	...	...	...
*N*+1	N&D	core-hole plus *p*′_2_→*p*_2_	*E_b_* + (ε_*p*_1__–ε_*p*′_2__)	γ/2	*V*_**p**_2_,**p**′_2__		
	CR	resonance configuration *N*+1	*E*_*N*+1_	Γ_*N*+1_	*V*_1,*N*+1_	*V*_2,*N*+1_	*V*_3,*N*+1_
*N*+2	N&D	core-hole plus *p*′_2_→*p*_2_	*E_b_* + (ε_*p*_2__–ε_*p*′_2__)	γ/2	*V*_**p**_2_,**p**′_2__		
	CR	resonance configuration *N*+2	*E*_*N*+2_	Γ_*N*+2_	*V*_1,*N*+2_	*V*_2,*N*+2_	*V*_3,*N*+2_
...	...	...	...	...	...	...	...

a The lifetime width is the same for all the states under the D&S approach. The numbering of the states is done by first considering transitions from valence band state *p*′1 to all *p_i_* states, following by *p*′_2_→*p_i_* transitions, and so on.

The D&S approach reduces to the CR approach when only a few excited valence band states are considered. Essentially, for a countable number of valence excitations, the CR approach handles explicitly the N&D Hamiltonian along with the lifetime effects that are implicit in the approach of D&S. This leaves open still the possibility of an “infrared catastrophe” if *N* approaches the macroscale, which would yet bring with it the lack of integrable line shape. Therefore, this casts doubt on whether *N* is ever so large, given that the asymmetry can be explained with an integrable form with fewer resonances in the CR approach.

## 3 Experimental and Analysis Details

### 3.1 Analyzed Materials

A Ta 4f X-ray photoelectron spectrum was downloaded from the XPSLibrary.^13^ It was acquired using a Quantum 2000 PHI system with an S-probe system (Surface Science Instruments) equipped with a monochromatic Al Kα source from 99.99% pure tantalum. The pass energy was set to 14.3 eV with 68 scans recorded and a dwell time of 0.1 s; constant analyzer energy (CAE) mode was employed. The operating pressure was 1.6 × 10^–9^ Torr. Additional experimental details are available in reference^14^.

The graphene sample used for the C 1s analysis corresponds to chemical vapor deposited (CVD) graphene grown on copper foils, with experimental data taken directly from the work of Pirkle et al.^15^ The analyzed sample is a continuous monolayer graphene with grain sizes of 10–20 μm, obtained by exposing copper foils to 5 sccm of hydrogen and 7 sccm of methane at a total pressure of 400 Torr in a halogen-lamp quartz tube furnace operated at 1035 °C. As demonstrated by Pirkle et al.,^15^ this preparation yields a clean graphene-on-Cu surface. The XPS spectra were collected using a monochromatic Al Kα source and an Omicron EA125 hemispherical analyzer, with an acceptance angle of ± 6°, a takeoff angle of 45°, and a pass energy of 15 eV.^15^

### 3.2 Peak-Fitting Analysis

Coupled-Resonances (CR) lineshapes Type-II and Type-III were used to model the asymmetry of the main peak in the aforementioned photoemission spectra. The Gaussian widths and all CR parameters of the main peak were optimized during fitting. Additional peaks were modeled using Voigt line shapes. Parameters such as peak centers and widths were treated as free variables, with their optimized values provided in the tables of the Supporting Information. In addition to the Ta 4f doublet, the spectrum also captures the 5p_3/2_ orbital, which was modeled using a Voigt peak. The 5p_1/2_ component lies at approximately ∼40 eV outside the energy range of the high-resolution spectrum.^16^ The complete set of peak parameters needed to reproduce the experimental spectra is found in the Supporting Information.

For doublets, the branching ratios were fixed to their corresponding Scofield cross sections.^17^ The Gaussian widths were optimized but constrained to be the same for both spin–orbit branches. The total background was modeled through the Active Approach,^18^ which combines multiple background types and optimizes their contributions during fitting. Specifically, the total background combines extrinsic, intrinsic, and baseline components. The extrinsic background was fitted using the 2-Parameter Tougaard background^19^ for the C 1s spectrum and the Tougaard background^20^ for the Ta 4f spectrum. In the case of Ta 4f, the Tougaard background was calculated using the material loss function obtained from reference^21^. In both cases, the intrinsic contribution to the background was modeled through the Narrow-Shirley approach.^22^

Uncertainties of the peak and background parameters were robustly determined by accounting for the covariances between each pair of parameters, including those of the background. This is possible through the covariant matrix method,^12^ which is encompassed in the software AAnalyzer.^9^ All the uncertainties reported correspond to one time the standard deviation.

Careful use of the CR lineshapes should be made, as with any other line shape, to avoid incorrectly modeling them with peaks corresponding to other chemical species.

## 4 Results and Discussion

A large fraction of the peaks in spectra from elements other than metals and metalloids from the third, fourth, and fifth rows of the Periodic Table are symmetric and can be modeled with Voigt distributions (Voigt corresponds to Type-I CR lineshapes, with N = 1 and Im|X_1_| = 0 in eq 15). However, many spectra present significant challenges due to pronounced peak asymmetries. This section showcases three particularly relevant examples that illustrate such complexities, both due to the widespread use of the materials and the highly asymmetric nature of their signals. The first example is the C 1s spectrum from graphene, modeled with a Type-II CR line shape for the main signal. The second is the Ta 4f photoemission spectrum from pure metallic tantalum, using a CR Type-III line shape. Contrast with other lineshapes is provided in Supporting Information Section. All fits are available at the XPS Oasis Platform.^23^

Crucially, before assigning a line shape model to a given peak, care was taken to verify whether the observed features were associated with oxide, suboxide, or other chemical species, especially due to the high reactivity of many metals. Only after ruling out these contributions could the peaks be confidently attributed to metallic resonances and modeled with a single-envelope CR line shape. This rigorous approach was consistently applied to all spectra analyzed, ensuring that the physical interpretation remained grounded in the actual chemical environment. These procedures underscore the importance of using CR lineshapes judiciously, as any other line shape, because misassignments could lead to incorrect conclusions about the electronic structure of the material.

### 4.1 C 1s from Graphene

For the C 1s spectrum from graphene (digitized from reference^15^), four fitting approaches for the main peak are presented in the Supporting Information. These include: (i) two Voigt peaks; (ii) a CR Type-II peak; (iii) a Double-Lorentzian peak; and (iv) one asymmetric Doniach-Sunjic peak. The fits obtained using CR Type-III and Type-II lineshapes are very similar; however, the CR Type-II model is preferred due to its lower number of fitting parameters. Among all approaches, the CR Type-II line shape—shown in Figure 5a—provides the most satisfactory results. It effectively captures the asymmetry of the main peak with a small structure in the residual signal around the tip. The resulting energy positions and lifetime widths of the two resonances, along with the corresponding background fitting parameters, are listed in Table 2.

**Figure 5:**
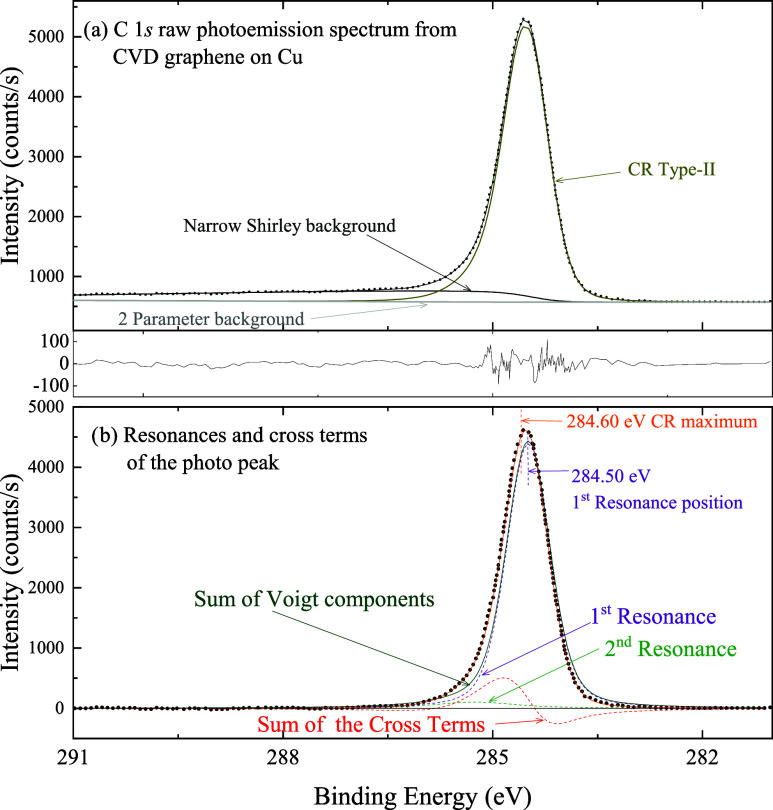
(a) Peak-fitting of the C 1s core level photoemission spectrum from graphene, using a CR Type-II line shape for the photo peak (Table 2 of the Supporting Information displays the corresponding fitting parameters). (b) Main peak and its CR components: the two symmetric components (resonances), their sum, and the sum of the cross terms. For clarity and to facilitate visual identification, only the sum of the cross terms is shown. The experimental data is shown after background subtraction (a 2-Parameters Tougaard Background and a Narrow Shirley background with parameters given in Table A2 of the Supporting Information).

**Table 2:** Eigenvalues and X-Parameters of the Main Peak for C 1s in Graphene

*E*_*R*_1__ (eV)	Γ_*R*_1__ (eV)	*E*_*R*_2__ (eV)	Γ_*R*_2__ (eV)
284.5 ± 0.1	0.2 ± 0.1	285.2 ± 0.1	1.13 ± 0.61
Re[X_1_]	Im [X_1_]	Re[X_2_]	Im [X_2_]
0.95 ± 0.12	–0.14 ± 0.06	0.05 ± 0.12	0.14 ± 0.06

As in the case shown in Figure 3, the sum (solid green line in Figure 5b) of the two symmetric Voigt functions associated with resonances 1 and 2 (dashed purple and green lines in Figure 5b) does not reproduce the experimental data. The asymmetry of the main signal is not only caused by the presence of two resonances, but mainly by the cross terms in eq 15 (dashed red curve in Figure 5b): while the region where it is negative causes an abrupt rise on the low-binding-energy side of the peak, the range in which it is positive—peaking at ∼285 eV—produces a more gradual decay on the high-binding-energy side. Moreover, the cross terms shift the position of the peak maximum toward higher binding energy. Note that the maximum of the CR Type-II curve (orange vertical line in Figure 5b) does not coincide with either the main resonance position (purple vertical line) or with the maximum of the sum of the resonances.

Figure 5b was generated by subtracting the total background from the experimental data shown in Figure 5a. The total background, as mentioned in Section 2.2, combines the 2-Parameter Tougaard and the Narrow-Shirley backgrounds (parameters listed in Table A2 of the Supporting Information). The main peak is constituted by two Voigt distributions, one per resonance, and the cross-terms described in eq 15.

- Voigt Components: These are calculated by first determining the Lorentzian component (using the first term of eq 15) and then convolving it with a Gaussian line shape. The Gaussian width is set to the value reported for peak 0 in Table A2. The sum of the two Voigt functions (dashed green lines in Figure 5b) does not accurately represent the experimental data, highlighting the importance of the cross terms.
- Cross-Term Components: These are calculated using the second term of eq 15 and are similarly convolved with the same Gaussian line shape. The envelope of the total cross terms is the sum of the individual cross-term components.

An interesting finding from Table 2 is the difference in intensity relationships between the two resonances. For the first resonance, the real part of the complex intensity is almost 7 times larger than its imaginary part. Conversely, for the second resonance, the imaginary part is about 3 times larger than the real part. This is noteworthy because the cross-term of the smaller second resonance is equal in magnitude to that of the main resonance.

### 4.2 Ta 4f from Metallic Tantalum

This is an example of very asymmetric peaks. The Supporting Information (Section A3) shows five different approaches for fitting the main signal of the Ta 4f spectrum. These include: (i) multiple symmetric peaks (requiring five components), (ii) combining one asymmetric peak CR Type-II with a Voigt, (iii) a single CR Type-III line shape, (iv) a double-Lorentzian line shape combined with a symmetric peak, and (v) employing one asymmetric Doniach–Šunjić peak with two symmetric peaks.

Among these, the CR Type-III line shape provided the most satisfactory fit (Figure 6); the associated eigenvalues and complex intensities are displayed in Table 3. It accurately reproduces the intricate features of the main signal without requiring additional peaks. In particular, it captured the sharp rise on the low-binding energy side and the slower decay on the high-binding energy side of the two spin–orbit branches. Although the third resonance has a smaller real component, its imaginary counterpart exhibits a significant contribution. The energies and lifetime widths of the three resonances can, in principle, be compared with ab initio calculations (there are no reports in the literature).

**Figure 6:**
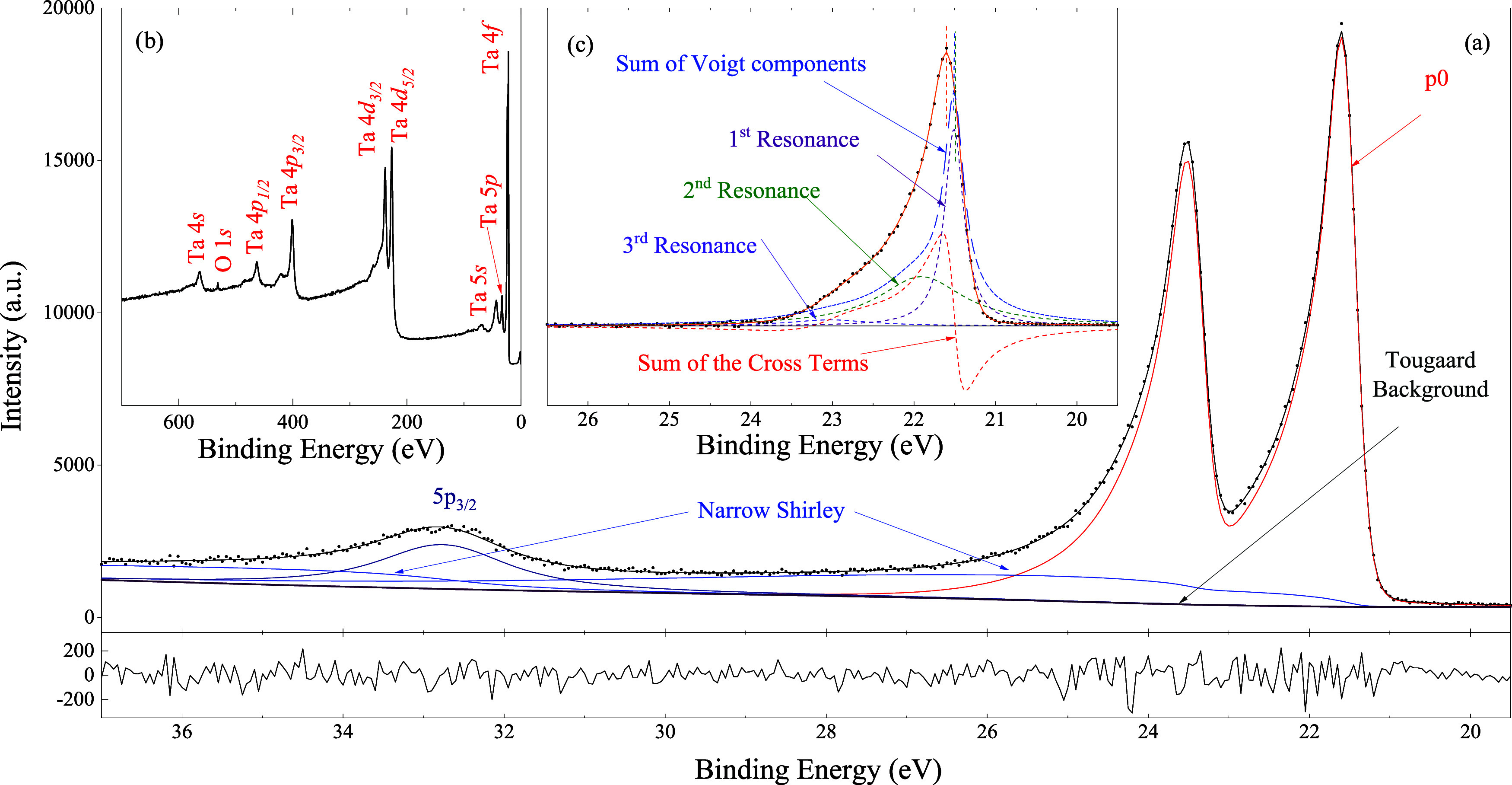
(a) The fit of the asymmetric Ta 4f core level photoemission spectrum from metallic tantalum has no peaks associated with its oxide. Table 3 displays the resonance’s eigenvalues and complex intensities; the fitting parameters are shown in the Complementary Material. The inelastic cross-section, obtained from reference^21^, was employed to assess the Tougaard background. The insert at the bottom shows the residual difference between the experimental data and the fitted curve, which exhibits no discernible structure, confirming the quality of the fit. (b) The small O 1s signal in the survey corresponds to a negligible amount of tantalum oxide (∼1.6 × 10^–2^ monolayers). (c) The signal corresponding to the 4f_7/2_ branch was obtained by subtracting the 4f_5/2_ component and the background. The negative part of the interference terms generates a sharp rise in the signal at the high binding energy side of the spectrum. The interference terms shift the signal with respect to the maximum of the peaks associated with the resonances. This effect, the “CR shift”, is usually neglected in the calculation of the total signal from multiplet components.

**Table 3:** Eigenvalues and X Factors of the Main Peak of the Ta 4f Photoemission Spectrum from Metallic Tantalum

branch	*E*_*R*_1__ (eV)	Γ_*R*_1__ (eV)	*E*_*R*_2__ (eV)	Γ_*R*_2__ (eV)	*E*_*R*_3__ (eV)	Γ_*R*_3__ (eV)
p0 4f_7/2_	21.50 ± 0.80	0.30 ± 5.30	21.90 ± 3.50	1.27 ± 6.22	23.03 ± 3.86	1.22 ± 2.92
p0 4f_5/2_	23.40 ± 1.10	0.40 ± 2.20	23.90 ± 3.30	1.77 ± 3.33	25.57 ± 2.51	4.17 ± 3.32
	Re[X_1_]	Im[X_1_]	Re[X_2_]	Im[X_2_]	Re[X_3_]	Im[X_3_]
p0 4f_7/2_	0.44 ± 2.14	–0.4 ± 6.23	0.5 ± 2.6	0.2 ± 8.1	0.06 ± 0.69	0.20 ± 1.90
p0 4f_5/2_	0.51 ± 2.16	–0.5 ± 2.7	0.57 ± 2.74	0.3 ± 2.8	–0.08 ± 0.69	0.20 ± 0.60

The tantalum 4f survey spectrum shown in panel (b) of Figure 6 exhibits a small O 1s peak. The MultiLayer Method^24^ was applied to estimate the thickness of the related tantalum oxide, resulting in a negligible amount (∼1.6 × 10^–2^ monolayers). This is consistent with the absence of peaks associated with tantalum oxides such as Ta_2_O_5_ (∼26.5 and 28.4 eV).

### 4.3 Application to Other Materials

The versatility of the Coupled-Resonances (CR) line shape is evident in its successful application to every photoemission spectrum with asymmetric peaks that we analyzed. This comprehensive applicability includes studies such as the analysis of Fe 2p spectra in hematite (Fe_2_O_3_) and magnetite (Fe_3_O_4_), which provided valuable insights by allowing us to compare the resonance energies and lifetimes with multiplet calculations.^25^ Moreover, by applying the CR model to the 3d spectra of fourth-row elements, we were able to identify and elucidate systematic trends in the energy separation between resonances and their lifetime widths, further highlighting the model’s analytical power.^26^

## 5 Conclusions

This work addresses the long-standing challenge of accurately modeling asymmetric peaks in photoelectron spectroscopy, which are prevalent across a wide range of materials from metals to oxides. We introduce the Coupled-Resonances (CR) line shape as a theoretically robust and versatile model that extends the conventional Lorentzian distribution by incorporating quantum interference effects when the initial state, right after the photoelectron is ejected, coexists in various resonance states. This innovative approach overcomes the limitations of the widely used Doniach-Šunjić (D&S) model, particularly its nonintegrability and narrow applicability to only metallic systems. The CR approach correctly predicts that asymmetries are expected for *p*, *d*, and *f*-type core levels in materials with open *p*, *d*, and *f*-type valence bands.

The CR framework accurately describes peak asymmetry by considering the nonorthogonality of coupled resonance eigenstates, which arises from a non-Hermitian Hamiltonian. The familiar symmetric Lorentzian line shape is a natural simplification of our model (CR Type I), while CR Type II and III effectively account for additional resonances associated with varying energies from different valence electron configurations within the core-hole potential.

It is shown that the total intensity is not only the sum of the peaks associated with each resonance, but extra interference terms play a crucial fundamental role in the asymmetry of the peak. A visible consequence is that these terms shift the maxima of the peak away from the maxima of the symmetric resonance contributions. This invites a revision of the way approaches using multiplet analysis assess the total line shape.

A significant practical advantage of the CR model is its implementation in the freely available AAnalyzer software, offering an efficient and streamlined peak-fitting process that requires fewer peaks and operates with surprising speed compared to traditional methods. The broad applicability and robustness of the CR model were demonstrated across diverse experimental spectra, including metals, metalloids, metal oxides, and graphene. In all cases, the CR type II or III lineshapes enabled accurate reproduction of the asymmetric main peaks while allowing the extraction of physically meaningful parameters, such as resonance energies and lifetimes, that are often inaccessible through traditional fitting approaches.

The analysis of the Ta 4f spectrum using CR type-III showed that it is possible to model both spin–orbit branches with a single peak envelope, capturing sharp features that are difficult to reproduce with conventional methods. In the graphene C 1s spectrum, CR type-II provided a stable and minimal-parameter fit that resolved the asymmetry and allows for additional components when necessary.

Overall, the CR framework emerges as a robust, generalizable, and physically grounded tool for modeling core-level asymmetries, improving upon both empirical fitting and traditional peak-decomposition strategies. It enhances reproducibility, enables cross-sample comparisons, and bridges the gap between high-level theory and experimental data analysis.

Future work will aim to further refine the model for better extraction of Hamiltonian-related quantities and extend its application to broader spectral databases. The framework also shows promise for adaptation to X-ray and optical fluorescence spectra, paving the way for more comprehensive and physically grounded spectral analysis across diverse experimental techniques.

## Supplementary Material



## Notes

Comparison of CR line shape to other lineshapes, demonstration of eq 15 (PDF)
