# Supplementary material for: The Coupled-Resonances Asymmetric Lineshape for Photoemission Spectra
Source: J Phys Chem C Nanomater Interfaces. 2025 Nov 17;129(47):21162–73. doi: 10.1021/acs.jpcc.5c05560 (PMC12670491; doi:10.1021/acs.jpcc.5c05560)
Supplement: Supplementary file 1 [file jp5c05560_si_001.pdf]

Supplementary Material for the paper titled:

The Coupled-Resonances Asymmetric Lineshape for Photoemission Spectra

Alberto Herrera-Gomez,[1] Dulce Maria Guzman-Bucio,[1] Dagoberto Cabrera-German,[2] Abraham Carmona-Carmona,[3] Vincent Crist,[4] Anthony D. Dutoi[5]

[1]CINVESTAV-Unidad Queretaro, Queretaro, 76230, Mexico
[2]Departamento de Investigación en Polímeros y Materiales, Universidad de Sonora, Hermosillo, 83000, Mexico
[3]Benemérita Universidad Autónoma de Puebla, Puebla, 72000, Mexico
[4]The XPS Library, Salem, Oregon 9730, USA.
[5]Department of Chemistry, University of the Pacific, Stockton, CA, USA

1 Comparison of the CR lineshape to other lineshapes

1.1 Overview

Most peaks in spectra from elements other than metals and metalloids from the 3rd, 4th, and 5th rows of the Periodic Table can typically be modeled using Voigt distributions. Voigt corresponds to Type I CR lineshapes (only one resonance). This section focuses on representative spectra (C 1*s* from graphene and Ta 4*f* from metallic tantalum) with peaks requiring Type II and -III CR lineshapes. Each figure in this section is accompanied by a table presenting the fitting parameters, along with the resulting energies and lifetime broadenings of the core-hole configurations extracted from the eigenvalues of the Hamiltonian. These values could eventually be compared with results from first-principles calculations.

In spectra exhibiting asymmetric peaks, the asymmetry is generally limited to the main peak, while the remaining peaks are usually symmetric (Voigt) and dominated by their Gaussian components. For comparison purposes, the fits presented in this section employ the following approaches to model the main (asymmetric) peak:

A) Set of symmetric Voigt (i.e., Type I CR) peaks.
B) Type II CR in combination with symmetric peaks.
C) Type III CR (when required) in combination with symmetric peaks.
D) Double Lorentzian in combination with symmetric peaks.
E) Doniach Sunjic in combination with symmetric peaks.

In the examples below, these labels (A, B, C, D, and E) are used within the text and figures to refer to the corresponding fitting approaches both within the text and the plots. For spectra where a Type II CR adequately reproduces the asymmetry of the main peak, Approach C is not discussed. In the tables displaying the parameters, the lineshape is described by a letter: "V" for Voigt, "G" for Gaussian, "CR II" for CR Type II, "CR III" for CR Type III, "DL" for Double Lorentzian,

"DS" for Doniach-Sunjic. The background is modeled through a combination of Narrow-Shirley[1] and Tougaard[2] backgrounds.

1.2 C 1*s* photoemission spectrum from graphene

The peak-fitting analysis for a graphene C 1*s* spectrum is presented in Figure A1, where different fitting approaches—A, B, D, and E—are employed. The analysis includes a vertically expanded view of the peak-fitting (right panels) to provide a more detailed assessment. The resultant peak areas with the different fitting approaches are displayed in Table A5. The complete set of peak-fitting parameters for each approach is provided in Tables A1 to A4.

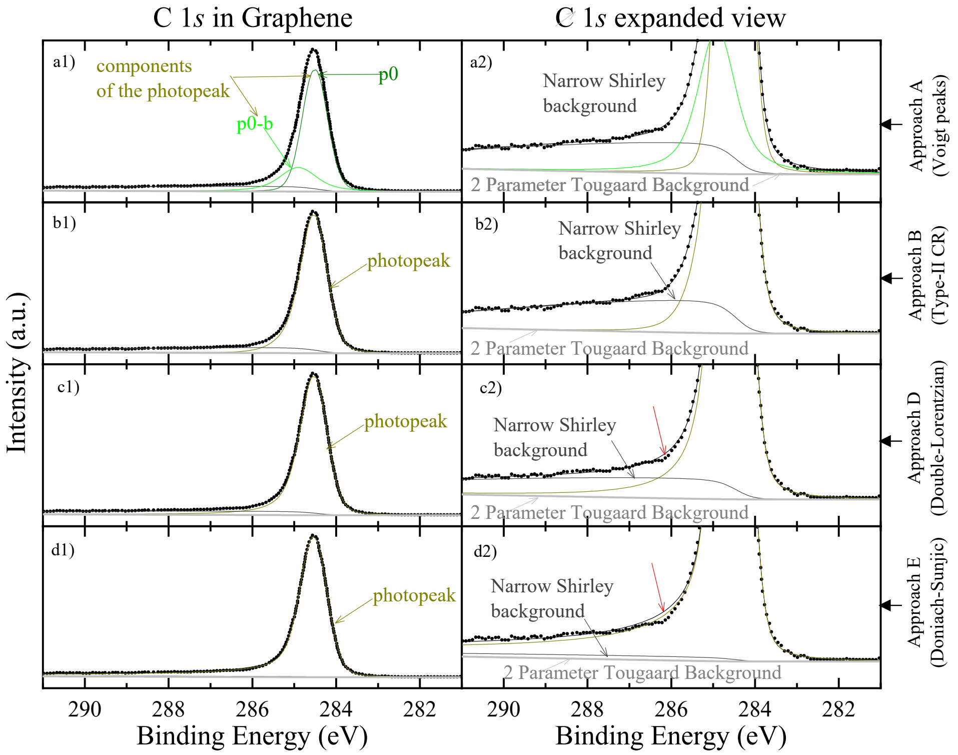

Figure A1. Comparison of fittings of a C 1*s* core level photoemission spectrum from CVD graphene on Cu employing different lineshapes to reproduce the asymmetry of the main peak. Tables A1 to A4 show the peak-fitting parameters for each approach.

1.2.1 Approach A: fitting with Voigt lineshapes

In Approach A (Figure A1-a), the spectrum is accurately reproduced using two Voigt profiles, requiring five free parameters (see Table A1; to stabilize the fit, the Gaussian width of peaks p0-b was constrained to match that of p0-a, an assumption that equates the instrumental broadening across the two symmetric components. In this way, a total of six parameters can be optimized.

This method lacks physical constraints on asymmetry: the relative binding energy, intensities, or widths of peaks p0-a and p0-b serve purely to reproduce the lineshape and are not tied to a predictive model.

Table A1. Peak fitting parameters of C 1*s* photoemission spectrum with approach A (Voigt).

Line Shape	Peak	Binding energy (eV)	Gaussian width (eV)	Lorentzian width (eV)	Relative area
Voigt	p0-a	284.50±0.01	0.65±0.01	0.08±0.05	74.0±0.2
Voigt	p0-b	284.90±0.10	Correlated to p0-a	0.62±0.08	26.0±0.2
Background parameters					
B-2 (eV^2)	C-2 (eV^2)	s_{NS} (eV^{-1}) (for p0-a and -b)	T_w (eV) (for p0-a and -b)	σ_d (eV) (for p0-a and -b)	Baseline (c/s)
2866 (fixed)	1640 (fixed)	0.040±0.002	0.2 (fixed)	8.45±0.77	556±4

1.2.2 Approach B: fitting with a Type II CR lineshape

Approach B, based on the Type II CR model, also utilizes 6 free parameters (Table A3) and achieves an excellent fit (see Figure A1-b). This approach employs only one asymmetric peak to reproduce the experimental spectrum fully. It is worth mentioning that the main peak area obtained with approach B differs only by ~4% from that of approach A (Table A5), indicating a consistent description of the dominant peak, reinforcing its reliability for chemical quantification.

A key advantage of CRII is that it avoids the excessive peak overextension observed in Doniach-Sunjic-based approaches,[3] which can distort background fitting and lead to misinterpretations of the surface chemical states. This characteristic makes CRII more representative of different chemical species at the surface, as it prevents artificial spectral broadening. Moreover, the CR approach enables a more reliable chemical composition by ensuring that the asymmetric peak does not extend beyond the expected binding energy range. Unlike asymmetric Voigt-based models, which frequently misrepresent satellite intensities and carbonaceous specie contributions, CR fits provide a more stable and physically meaningful peak decomposition, reinforcing their suitability for graphene and carbonaceous material analysis.

Table A2. Peak fitting parameters of the C 1*s* photoemission spectrum with approach B (CR Type II) for the photo peak. CR stands for Coupled-Resonances.

Line Shape	Binding energy (eV)	Gaussian width (eV)	ΔE_{12} (eV)	Γ_1 (eV)	Γ_2 (eV)	V_{12} (eV)
CR II	284.60±0.01	0.60±0.03	-0.55±0.08	0.06±0.07	1.34±0.66	0.34±0.10

Background parameters					
B-2 (eV^2)	C-2 (eV^2)	s_{NS} (eV^{-1})	T_w (eV)	σ_d (eV)	Baseline (c/s)
2866 (fixed)	1640 (fixed)	0.043±0.001	0.2 (fixed)	7.3±0.5	571±4

Resonances' energies, lifetime widths, and complex intensities

E_{R_1} (eV)	Γ_{R_1} (eV)	E_{R_2} (eV)	Γ_{R_2} (eV)
284.50±0.10	0.22±0.10	285.25±0.11	1.17±0.63
Re[X_1]	Im [X_1]	Re[X_2]	Im [X_2]
0.95±0.12	-0.14±0.06	0.05±0.12	0.14±0.06

1.2.3 Approach D: fitting with a Double-Lorentzian lineshape

The Double-Lorentzian approach (Approach D, Figure A1-c) further reduces the number of free parameters for the main signal to 4 (Table A3), simplifying the fitting process. However, this comes at the cost of a lack of physical interpretation of the fitting parameters. Nevertheless, this approach closely resembles previous literature reports[3–6] making it a practical alternative for fitting without excessive parameterization.

Table A3. Peak fitting parameters of the C 1*s* photoemission spectrum with approach D (Double-Lorentzian). DL stands for Double-Lorentzian.

Line Shape	Binding energy (eV)	Gaussian width (eV)	Lorentzian width (eV)	DL parameter
DL	284.44±0.01	0.59±0.01	0.17±0.01	2.34±0.13

Background parameters					
B-2 (eV^2)	C-2 (eV^2)	s_{NS} (eV^{-1})	T_w (eV)	σ_d (eV)	Baseline (c/s)
2866 (fixed)	1640 (fixed)	0.030±0.001	0.2 (fixed)	10.1±1.3	565±4

1.2.4 Approach E: fitting with a Doniach-Šunjić lineshape

Finally, Approach E, based on the Doniach-Šunjić model (Figure A1-d), requires 4 free parameters for the main signal (Table A4) but at the cost of a poor fit. While DS models inherently account for asymmetry, they frequently result in overextension toward higher binding energies, distorting background fitting and leading to inconsistencies in oxidation state identification. This is consistent with previous findings[3,6] about the Doniach-Sunjic models tending to require additional peaks, deviating from the expected spectral representation. This suggests that, while the Doniach-Sunjic function accounts for asymmetry, it may not fully describe the intrinsic asymmetry of the C 1*s* peak in graphene. It is noteworthy that the total area differs greatly (24%, Table A5) from the results obtained using Voigt profiles, making it less reliable for precise chemical quantification.

Table A4. Peak fitting parameters of the C 1*s* photoemission spectrum with approach E (Doniach-Šunjic). DS stands for Doniach-Šunjić.

Line Shape	Binding energy (eV)	Gaussian width (eV)	Lorentzian width (eV)	α
DS	284.50±0.01	0.66±0.01	0.12±0.01	0.090±0.002

Background parameters					
B-2 (eV^2)	C-2 (eV^2)	s_{NS} (eV^{-1})	T_w (eV)	σ_d (eV)	Baseline (c/s)
2866 (fixed)	1640 (fixed)	0.0013±0.0003	0.2 (fixed)	10 (fixed)	565±4

1.2.5 Conclusions for the C 1*s* analysis

Table A5 summarizes the resulting main photoemission signal areas obtained using the different fitting approaches as well as the number of free parameters.

The fitting approach using the Double-Lorentzian (DL) lineshape uses a lower number of optimizable parameters compared to the Voigt or CR Type II lineshapes. The area derived from the DL fit is within 3% difference of the Voigt area and is 3% larger than the CR Type II area. However, it is currently not possible to assign a physical meaning to the DL parameters.

Conversely, the approach using the Doniach-Sunjić (DS) lineshape clearly overestimates the experimental data upon close inspection. Furthermore, the fitted peak signal does not return to the baseline within the analyzed region, which is physically unrealistic. Consequently, the calculated area within the analyzed region is significantly larger than that obtained from the other approaches.

Table A5. Photopeak areas for the C 1s spectrum from CVD graphene on Cu using different lineshapes

Photopeak area	Lineshape			
	Voigt	CR II	Double-Lorentzian	Doniach-Šunjić
Counts/s	4349.2	4179.6	4478	5392
Normalized to the CRII area %	104	100	107	129
Free parameters	5	6	4	4

The CR Type II lineshape provides the best fit for the C 1s photoemission signal from graphene. It accurately reproduces both the abrupt rise in intensity on the lower binding energy side and the subsequent initial abrupt, then slow, decrease in intensity on the higher binding energy side. The area yielded by this approach is within 4% of the area obtained using Voigt lineshapes.

1.3 Ta 4*f* photoemission spectrum from metallic tantalum

Figure A2 shows the results from using Approaches A-E to fit the Ta 4*f* photoemission spectrum from pure metallic tantalum. In most cases, except for Approach C, more than two doublets are required to fit the main signal satisfactorily. The fitting parameters are given in Tables A6 through A10.

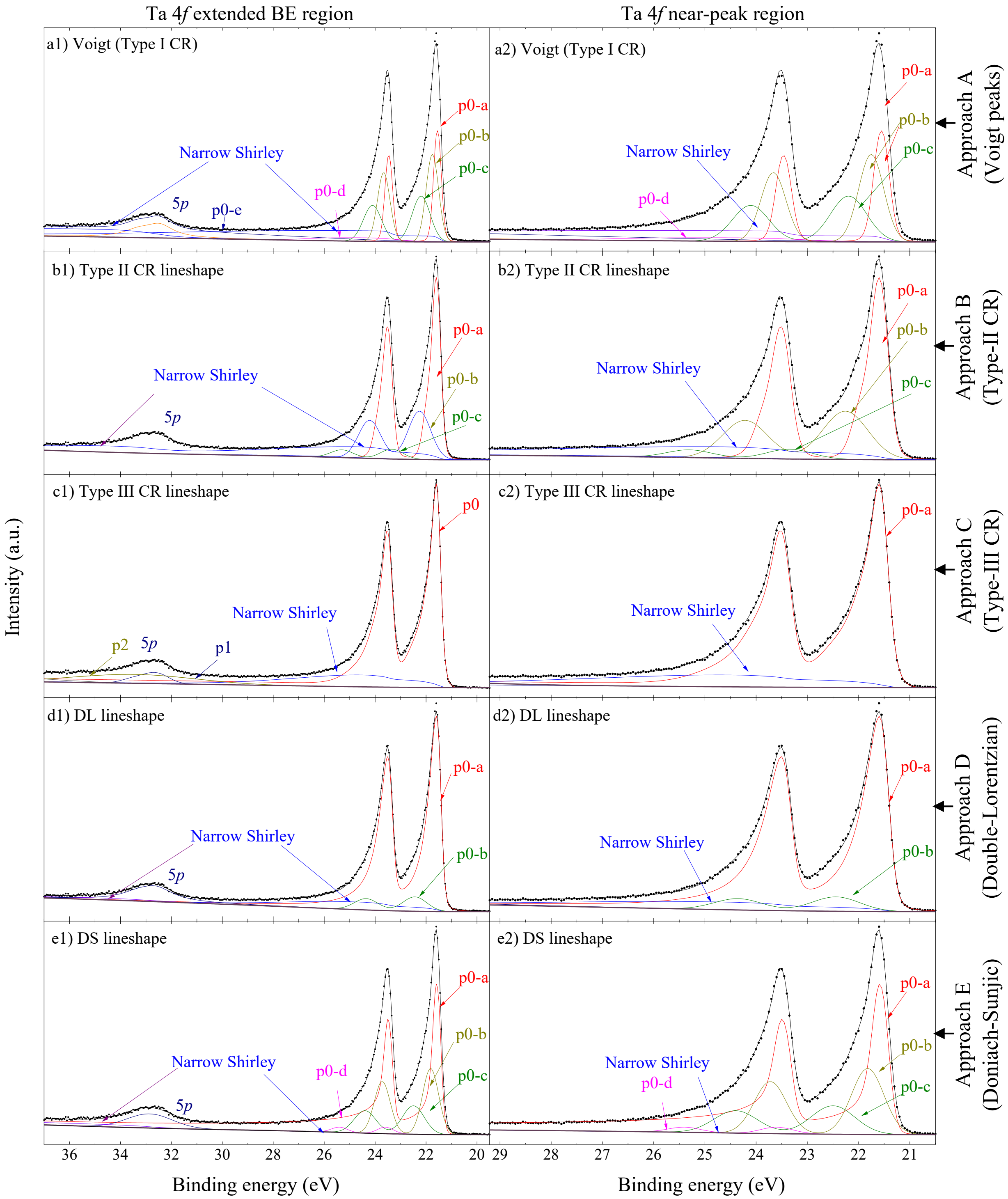

Figure A2. Fit of the asymmetric Ta 4*f* core level photoemission spectrum of metallic tantalum. Comparison of different lineshapes and their ability to reproduce the observed asymmetry. See Table A6-Table A10 for fitting parameters. The background is modeled through a combination of Narrow-Shirley and Tougaard backgrounds; the Tougaard background was modeled for all fits by deconvoluting the total spectrum with the experimentally determined normalized inelastic cross section obtained from Reference [7].

1.3.1 Approach A: fitting with Voigt lineshapes

Fitting the Ta 4*f* core-level spectrum with Approach A (Voigt lineshapes) presented significant challenges, particularly around 21 eV, where the $4f_{7/2}$ peak component begins. This region's sharp slope increase (Figure A2-a2) proved difficult to model accurately. While a combination of Voigt and Gaussian lineshapes with additional components was employed, this resulted in an excessive number of potentially non-physical peaks and still failed to model the spectrum adequately. This fit required 22 parameters, 15 of which were free, while the remaining 7 were fixed for model stability (Table A6).

Table A6. Fitting parameters of Ta 4*f* photoemission spectrum with approach A (Voigt). The branching ratio of the Ta 4*f* doublet was fixed at 0.7883. The divided cells correspond to the 7/2 (upper) and 5/2 (lower) spin-orbit branches. A Tougaard background was modeled by deconvoluting the total spectrum with the experimentally determined normalized inelastic cross section obtained from Reference [7]. "V" stands for Voigt and "G" for Gaussian.

<table>
<tr><th>Peak</th><th>Line Shape</th><th>Binding energy (eV)</th><th>Spin-orbit splitting (eV)</th><th>Gaussian width (eV)</th><th>Lorentzian width (eV)</th><th>s_{NS} (eV^{-1})</th><th>Relative main peak area</th></tr>
<tr><td>p0-a</td><td>V</td><td>21.59±0.13</td><td>1.9±0.01</td><td>0.31±0.01</td><td>0.02±0.01</td><td>0.126±0.003</td><td rowspan="2">43.2±0.1</td></tr>
<tr><td>p0-b</td><td>V</td><td>22.84±0.04</td><td>1.91 (Fix)</td><td>0.72±0.12</td><td>1.00±0.01</td><td>Correlated to p0</td></tr>
<tr><td>p0-c</td><td>G</td><td>22.19±0.01</td><td>1.91 (Fix)</td><td>0.86±0.02</td><td>-</td><td>Correlated to p0</td><td>23.9±0.1</td></tr>
<tr><td>p0-d</td><td>G</td><td>23.97±0.2</td><td>1.91 (Fix)</td><td>2.59±0.75</td><td>-</td><td>Correlated to p0</td><td>4.4±0.2</td></tr>
<tr><td>p0-e</td><td>G</td><td>21.75±0.2</td><td>1.91 (Fix)</td><td>0.56±0.15</td><td>-</td><td>Correlated to p0</td><td>28.3±0.1</td></tr>
<tr><td>$5p_{3/2}$</td><td>V</td><td>32.45±0.4</td><td>-</td><td>0.86±0.18</td><td>1.46±1.41</td><td>0.125±0.001</td><td></td></tr>
</table>

Background parameters

s_{NS} (eV^{-1}) (for p0)	T_W (eV) (for p0)	σ_d (eV) (for p0)	Baseline (c/s)
0.13±0.01	1.74 (Fixed)	10 (Fixed)	322.2±2.6

1.3.2 Approach B: fitting with a Type II CR lineshape

Approach B (CR type II) significantly reduced the number of peaks required for fitting the Ta 4*f* core level compared to the Voigt lineshape. Only two components were necessary. A Gaussian component was included to improve the fit to the slopes following the $4f_{7/2}$ and $4f_{5/2}$ peak maxima, where some data points deviated slightly (see Figure A2-b2). Fitting parameters are provided in Table A7.

Table A7. Fitting parameters of the Ta 4*f* photoemission spectrum with approach B (CR type II). The branching ratio of the Ta 4*f* doublet was fixed at 0.7883, and its spin-orbit splitting was found to be 1.98 ± 0.01 eV. The divided cells correspond to the 7/2 (upper) and 5/2 (lower) spin-orbit branches. A Tougaard background was modeled by deconvoluting the total spectrum with the experimentally determined normalized inelastic cross section obtained from Reference [7]. The s_{NS} parameter was correlated for all components of the main peak. The resulting eigenvalues of the Hamiltonian are indicated. CR II stands for Type II Coupled-Resonances, V for Voigt, and G for Gaussian.

Peak	Line Shape	Binding energy (eV)	Gaussian width (eV)	ΔE_{12} (eV)	Γ_1 (eV)	Γ_2 (eV)	V_{12} (eV)	Relative main peak area
p0-a	CRII	21.67±0.01	0.31±0.01	-0.219±0.004	0.03±0.01	0.28±0.05	0.19±0.01	61±0.1
	CRII	23.58±0.01	correlated to p0	-0.19±0.01	0.05±0.05	0.35±0.04	0.19±0.01	

Peak	Line Shape	Binding energy (eV)	Gaussian width (eV)	Lorentzian width (eV)	Relative area
p0-b	G	22.25±0.01	1.03±0.04	-	32±0.2
p0-c	G	23.34±0.03	Correlated to p0-b	-	7±0.1
$5p_{3/2}$	V	32.69±0.01	Correlated to p0-a	1.95±0.05	

Background parameters

s_{NS} (eV^{-1}) (for p0)	Tw (eV) (for p0)	σ_d (eV) (for p0)	Baseline (c/s)
0.069±0.017	1 (Fixed)	4.53±1.41	386.9±2.4

Resonance energies, lifetime widths, and complex intensities

Branch of the main peak	E_{R_1} (eV)	Γ_{R_1} (eV)	E_{R_2} (eV)	Γ_{R_2} (eV)
$4f_{7/2}$	21.56±0.01	0.21±0.01	22.09±0.01	0.04±0.04
$4f_{5/2}$	23.47±0.01	0.27±0.04	23.99±0.01	0.13±0.03
**Branch of the main peak**	**Re[X_1]**	**Im [X_1]**	**Re[X_2]**	**Im [X_2]**
$4f_{7/2}$	0.23±0.02	0.11±0.02	0.76±0.02	-0.11±0.02
$4f_{5/2}$	0.24±0.02	0.13±0.02	0.75±0.02	-0.13±0.02

1.3.3 Approach C: fitting with a Type III CR lineshape

The main signal of the Ta 4*f* photoemission spectrum exemplifies a case where Approach C (CR Type III) is essential. Using Approach B (CR Type II) still requires additional peaks to achieve an accurate fit. In contrast, Approach C (CR Type III) successfully resolves the entire spectrum with a single peak (Figure A2-c2), demonstrating its superiority for this case. This method precisely fits the $4f_{7/2}$ and $4f_{5/2}$ peak maxima, including the abrupt slope change in the rising region. It also accurately reproduces the slopes following the maxima, the subtle slope variations, and the characteristic Ta 4*f* peak asymmetry. Furthermore, Approach C optimizes the Narrow Shirley parameter. For the Ta 4*f* spectrum, the resonance lifetime widths remained positive. The parameters, calculated lifetimes, and eigenvalues for this approach are presented in Table A8.

Table A8. Fitting parameters of the Ta 4*f* photoemission spectrum with approach C (Type III CR lineshapes). The branching ratio of the Ta 4*f* doublet was fixed at 0.7883, and its spin-orbit splitting was found to be 1.98 ± 0.01 eV. The divided cells correspond to the 7/2 (upper) and 5/2 (lower) spin-orbit branches. A Tougaard background was modeled by deconvoluting the total spectrum with the experimentally determined normalized inelastic cross section obtained from Reference [7].The resulting eigenvalues of the Hamiltonian are indicated. CR III stands for Type III Coupled-Resonances and V for Voigt.

Peak	Line Shape	Binding energy (eV)	Gaussian width (eV)	ΔE_{12} (eV)	ΔE_{13}(eV)	Γ_1(eV)	Γ_2(eV)	Γ_3(eV)	V_{12}(eV)	V_{13}(eV)	V_{23}
$4f_{7/2}$	CRIII	21.97±0.01	0.22±0.01	-0.2±0.3	-0.3±0.1	0.17±0.14	-1.3±3.9	3.9±3.1	0.4±0.2	0.16±0.34	-1.5±1.6
$4f_{5/2}$	CRIII	24.05±0.01		0.3±0.8	-0.9±0.3	-0.2±0.8	0.4±2.8	6.1±1.5	0.6±0.2	0.8±1.1	-1.4±1.9
$5p_{3/2}$	V	32.74±0.01	0.6±0.7	-	-	-	-	-	-	-	-

Narrow-Shirley[1] background parameters

s_{NS} (eV^{-1}) (for p0)	*Tw* (eV) (for p0)	σ_d (eV) (for p0)	Baseline (c/s)
0.03+0.34	1 (Fixed)	7.7±0.6	340.1±3.3

Resulting real and imaginary parts of the CR Type III eigenvalues

Branch of the main peak	E_{R_1} (eV)	Γ_{R_1} (eV)	E_{R_2} (eV)	Γ_{R_2} (eV)	E_{R_3} (eV)	Γ_{R_3} (eV)
$4f_{7/2}$	21.50±0.80	0.30±5.30	21.90±3.50	1.27±6.22	23.03±3.86	1.22±2.92
$4f_{5/2}$	23.40±1.10	0.40±2.20	23.90±3.30	1.77±3.33	25.57±2.51	4.17±3.32

Resulting real and imaginary parts of the X_m Values

Branch of the main peak	Re[X_1]	Im[X_1]	Re[X_2]	Im[X_2]	Re[X_3]	Im[X_3]
$4f_{7/2}$	0.44±2.14	-0.4±6.2	0.5±2.6	0.2±8.1	0.06±0.69	0.20±1.90
$4f_{5/2}$	0.51±2.16	-0.5±2.7	0.57±2.74	0.3±2.8	-0.08±0.69	0.20±0.60

1.3.4 Approach D: fitting with a Double Lorentzian lineshape

Approach D (Double-Lorentzian) is similar to Approach B, as both require two peaks to fit the Ta 4*f* spectrum (Figure A2-d). Both also utilize a Gaussian lineshape for the second component. During fitting, a double Lorentzian lineshape was attempted for the second component, but the asymmetry consistently converged to one, confirming the appropriateness of the symmetric lineshape. The fitting parameters for Approach D are presented in Table A9.

Table A9. Fitting parameters of the Ta 4*f* photoemission spectrum with approach D (Double-Lorentzian). The branching ratio of the Ta 4*f* doublet was fixed at 0.7883. The divided cells correspond to the 7/2 (upper) and 5/2 (lower) spin-orbit branches. A Tougaard background was modeled by deconvoluting the total spectrum with the experimentally determined normalized inelastic cross section obtained from Reference [7]. The peak corresponding to 5*p* has an s_{NS} parameter of 0.146+001 eV^{-1}. DL stands for Double-Lorentzian, V for Voigt, and G for Gaussian.

Peak	Line Shape	Binding energy (eV)	Spin-orbit splitting (eV)	Gaussian width (eV)	Lorentzian width (eV)	DL parameter (eV)	Relative main peak area

p0-a	DL $4f_{7/2}$	21.46±0.001	1.92±0.01	0.230±0.005	0.11±0.02	7.81±0.16	91.8±0.1
	DL $4f_{7/2}$	23.38±0.001			0.13±0.02	7.15±0.27	
p0-b	G	22.48±0.01	1.91 (Fix)	1.10±0.01	-	-	8.1±0.1
$5p_{3/2}$	V	32.73±0.40	0.056 (Fix)	0.60±0.01	1.8±0.1	-	-

Background parameters

s_{NS} (eV^{-1}) (for p0)	Tw (eV) (for p0)	σ_d (eV) (for p0)	Baseline (c/s)
0.027±0.003	1 (Fixed)	6.1±0.2	366.0±2.5

1.3.5 Approach E: fitting with a Doniach-Šunjić lineshape

Approach E (Doniach-Sunjic) requires a large number of peaks to model the asymmetric Ta 4*f* spectrum. Even with this, the $4f_{7/2}$ and $4f_{5/2}$ peak maxima are not fully resolved. Figure A2-e2 shows that the slopes also poorly fit the experimental data, consistently deviating above or below the measured values. Furthermore, the shape of the Narrow Shirley component is unconventional, suggesting a lack of a clear physical basis. While Approach E is commonly used to fit asymmetric peaks, Approach C is significantly superior for the Ta 4*f* spectrum, reproducing the curve with 20 free parameters compared to the 25 required by Approach E. The fitting parameters for Approach E are presented in Table A10.

Table A10. Fitting parameters of the Ta 4*f* photoemission spectrum with approach E (Doniach-Šunjić). The branching ratio of the Ta 4*f* doublet was fixed at 0.7883. The divided cells correspond to the 7/2 (upper) and 5/2 (lower) spin-orbit branches. DS stands for Doniach-Šunjić, V for Voigt, and G for Gaussian.

Peak	Line Shape	Binding energy (eV)	Spin-orbit splitting (eV)	Gaussian width (eV)	α (eV)	Normalized peak area
p0-a	DS $4f_{7/2}$	21.52±0.01	1.90±0.01	0.31±0.01	0.21±0.02	64.7±0.1
	DS $4f_{7/2}$	23.42±0.01			0.22±0.41	
p0-b	G	21.82±0.02	1.89±0.01	0.74±0.01		20.9±0.1
p0-c	G	22.49±0.01	Correlated to p0-b	1 (Fix)		12.2±0.1
p0-d	G	23.59±0.01	1.80±0.01	Correlated to p0-b		2.0±0.1
$5p_{3/2}$	V	32.86±0.01	0.5 (Fix)	1.50±0.03		-

Background parameters

B-2 (eV^2)	*C-2* (eV^2)	s_{NS} (eV^{-1}) (for p0)	Tw (eV) (for p0)	σ_d (eV) (for p0)	Baseline (c/s)
-	1643 (Fixed)	0.0001±0.0020	15.01±1.41	1 (Fix)	387.65±2.24

1.3.6 Conclusions for the Ta 4*f* analysis

The peak areas corresponding to the different fitting approaches are summarized in Table A11. The analysis shows that Approach D (Double Lorentzian) requires a smaller number of parameters; this is at the cost of yielding parameters that cannot be related to physical quantities. Approach E (Doniach-Šunjić) yields a significantly larger peak area. The difference in area between CR II and CR III is mostly due to the contribution of the third interference term.

Table A11. Main peak area for the metallic Ta 4*f* spectrum using different lineshapes

Photopeak area					
Lineshape	Voigt (A)	CR II (B)	CR III (C)	Double-Lorentzian (D)	Doniach-Šunjić (E)
Counts/s	29951	27189	29153	30669	40757
Normalized to the CRIII area %	103	95	100	105	140
Free parameters	18	18	18	12	18

The CR Type III line shape provides the most accurate representation of the $4f_{7/2}$ and $4f_{5/2}$ components using a single peak. Unlike other line shapes, it effectively captures the spectral region around the peak maximum. Particularly notable is the ability of the CR Type III function to accurately reproduce the sharp slope change near 21 eV in the Ta 4*f* spectrum (Figure A2). This feature is exceptionally difficult to model, as the slope approaches a nearly vertical rise, which typically requires multiple overlapping components when using Voigt or DS line shapes. The efficiency of the CR Type III function in achieving this level of accuracy with parameters that can be related to physical quantities highlights its advanced modeling capability.

2 Demonstration of Equation 15 of the main text

Eq. 15 of the paper is the following:

$$\left|f_N(E)\right|^2 = \frac{1}{\pi}\sum_{m=1}^{N}\left[\mathrm{Re}(\mathrm{X}_m)\frac{\frac{\Gamma_{R_m}}{2}}{\left(E-E_{R_m}\right)^2+\left(\frac{\Gamma_{R_m}}{2}\right)^2}+\mathrm{Im}(\mathrm{X}_m)\frac{\left(E-E_{R_m}\right)}{\left(E-E_{R_m}\right)^2+\left(\frac{\Gamma_{R_m}}{2}\right)^2}\right]$$

Its demonstration goes as follows:

$$\langle\phi(0)|\phi(t)\rangle = \sum_{m=1}^{N} e^{-i\frac{\varepsilon_m}{\hbar}t}\left(\sum_{n=1}^{N}\alpha_n^*\alpha_m\mathbf{\Phi}_n^\dagger\mathbf{\Phi}_m\right) = \sum_{m=1}^{N} e^{-i\frac{\varepsilon_m}{\hbar}t}\mathrm{X}_m ,$$

$$\left|f(E)\right|^2 = \frac{1}{2\pi\hbar}\int_{-\infty}^{\infty} dt\, e^{i\frac{Et}{\hbar}}\langle\phi(0)|\phi(t)\rangle$$

$$\langle\phi(0)|\phi(t)\rangle = \begin{cases} \sum_{m=1}^{N} e^{-i\frac{\varepsilon_m^+}{\hbar}t}\,\mathrm{X}_m^+ & t<0 \\ \sum_{m=1}^{N} e^{-i\frac{\varepsilon_m^-}{\hbar}t}\,\mathrm{X}_m^- & 0\le t \end{cases}$$

$$|f(E)|^2 = \frac{1}{2\pi\hbar}\int_{-\infty}^{\infty} dt\ \exp\left(i\frac{E\,t}{\hbar}\right)\langle\phi(0)|\phi(t)\rangle$$

$$\langle\phi(0)|\phi(t)\rangle = \begin{cases} \sum_{m=1}^{N} \exp\left(-i\frac{\varepsilon_m^+}{\hbar}t\right)\mathrm{X}_m^+ & t<0,\ \text{with } \varepsilon_m^+ = E_m + i\frac{\Gamma_m}{2} \text{ and } \mathrm{X}_m^+ = \mathrm{X}_m^{\mathbb{R}} + i\mathrm{X}_m^{\Im} \\ \sum_{m=1}^{N} \exp\left(-i\frac{\varepsilon_m^-}{\hbar}t\right)\mathrm{X}_m^- & 0\le t,\ \text{with } \varepsilon_m^- = E_m - i\frac{\Gamma_m}{2} \text{ and } \mathrm{X}_m^- = \mathrm{X}_m^{\mathbb{R}} - i\mathrm{X}_m^{\Im} \end{cases}$$

$$|f(E)|^2 = \frac{1}{2\pi\hbar}\int_{-\infty}^{0} dt\,\exp\left(i\frac{E\,t}{\hbar}\right)\sum_{m=1}^{N}\exp\left(-i\frac{\varepsilon_m^+}{\hbar}t\right)\mathrm{X}_m^+ + \frac{1}{2\pi\hbar}\int_{0}^{\infty} dt\ \exp\left(i\frac{E\,t}{\hbar}\right)\sum_{m=1}^{N}\exp\left(-i\frac{\varepsilon_m^-}{\hbar}t\right)\mathrm{X}_m^-$$

$$= \frac{1}{2\pi\hbar}\sum_{m=1}^{N}\mathrm{X}_m^+\int_{-\infty}^{0} dt\,\exp\left(i\frac{E-\left(E_m + i\frac{\Gamma_m}{2}\right)}{\hbar}t\right) + \frac{1}{2\pi\hbar}\sum_{m=1}^{N}\mathrm{X}_m^-\int_{0}^{\infty} dt\,\exp\left(i\frac{E-\left(E_m - i\frac{\Gamma_m}{2}\right)}{\hbar}t\right)$$

The limits of the first integral can be inverted:

$$|f(E)|^2 = \frac{1}{2\pi\hbar}\sum_{m=1}^{N}\mathrm{X}_m^+\int_{0}^{\infty} dt\,\exp\left(i\frac{E-\left(E_m + i\frac{\Gamma_m}{2}\right)}{\hbar}(-t)\right) + \frac{1}{2\pi\hbar}\sum_{m=1}^{N}\mathrm{X}_m^-\int_{0}^{\infty} dt\,\exp\left(i\frac{E-\left(E_m - i\frac{\Gamma_m}{2}\right)}{\hbar}t\right)$$

Rearranging:

$$= \frac{1}{2\pi\hbar}\left[\sum_{m=1}^{N}\left(\mathrm{X}_m^{\mathbb{R}} + i\mathrm{X}_m^{\Im}\right)\int_{0}^{\infty} dt\,\exp\left(i\frac{-(E-E_m)+i\frac{\Gamma_m}{2}}{\hbar}t\right) + \sum_{m=1}^{N}\left(\mathrm{X}_m^{\mathbb{R}} - i\mathrm{X}_m^{\Im}\right)\int_{0}^{\infty} dt\,\exp\left(i\frac{(E-E_m)+i\frac{\Gamma_m}{2}}{\hbar}t\right)\right]$$

$$= \frac{1}{2\pi\hbar}\sum_{m=1}^{N}\mathrm{X}_m^{\mathbb{R}}\int_{0}^{\infty} dt\left[\exp\left(-i\frac{(E-E_m)}{\hbar}t\right) + \exp\left(i\frac{(E-E_m)}{\hbar}t\right)\right]\exp\left(-\frac{\Gamma_m}{2\hbar}t\right) +$$

$$+\sum_{m=1}^{N}\mathrm{X}_m^{\Im}\int_{0}^{\infty} dt\left[\exp\left(-i\frac{(E-E_m)}{\hbar}t\right) - \exp\left(i\frac{(E-E_m)}{\hbar}t\right)\right]\exp\left(-\frac{\Gamma_m}{2\hbar}t\right)$$

The integrals are straightforward:

$$=\frac{1}{2\pi\hbar}\sum_{m=1}^{N}\mathrm{X}_m^{\mathbb{R}}\left[\frac{1}{-i\frac{(E-E_m)}{\hbar}-\frac{\Gamma_m}{2\hbar}}\exp\left(-i\frac{(E-E_m)}{\hbar}t-\frac{\Gamma_m}{2\hbar}t\right)\Bigg|_{t=0}^{\infty}+\frac{1}{i\frac{(E-E_m)}{\hbar}-\frac{\Gamma_m}{2\hbar}}\exp\left(i\frac{(E-E_m)}{\hbar}t-\frac{\Gamma_m}{2\hbar}t\right)\Bigg|_{t=0}^{\infty}\right]+$$

$$\frac{i}{2\pi\hbar}\sum_{m=1}^{N}\mathrm{X}_m^{\Im}\left[\frac{1}{-i\frac{(E-E_m)}{\hbar}-\frac{\Gamma_m}{2\hbar}}\exp\left(-i\frac{(E-E_m)}{\hbar}t-\frac{\Gamma_m}{2\hbar}t\right)\Bigg|_{t=0}^{\infty}-\frac{1}{i\frac{(E-E_m)}{\hbar}-\frac{\Gamma_m}{2\hbar}}\exp\left(i\frac{(E-E_m)}{\hbar}t-\frac{\Gamma_m}{2\hbar}t\right)\Bigg|_{t=0}^{\infty}\right]$$

$$=-\frac{1}{2\pi\hbar}\sum_{m=1}^{N}\mathrm{X}_m^{\mathbb{R}}\left[\frac{1}{-i\frac{(E-E_m)}{\hbar}-\frac{\Gamma_m}{2\hbar}}+\frac{1}{i\frac{(E-E_m)}{\hbar}-\frac{\Gamma_m}{2\hbar}}\right]-\frac{i}{2\pi\hbar}\sum_{m=1}^{N}\mathrm{X}_m^{\Im}\left[\frac{1}{-i\frac{(E-E_m)}{\hbar}-\frac{\Gamma_m}{2\hbar}}-\frac{1}{i\frac{(E-E_m)}{\hbar}-\frac{\Gamma_m}{2\hbar}}\right]$$

$$=-\frac{1}{2\pi\hbar}\sum_{m=1}^{N}\mathrm{X}_m^{\mathbb{R}}\left[\frac{i\frac{(E-E_m)}{\hbar}-\frac{\Gamma_m}{2\hbar}-i\frac{(E-E_m)}{\hbar}-\frac{\Gamma_m}{2\hbar}}{\frac{(E-E_m)^2}{\hbar^2}+\frac{\Gamma_m^2}{(2\hbar)^2}}\right]-\frac{i}{2\pi\hbar}\sum_{m=1}^{N}\mathrm{X}_m^{\Im}\left[\frac{i\frac{(E-E_m)}{\hbar}-\frac{\Gamma_m}{2\hbar}-\left(i\frac{(E-E_m)}{\hbar}-\frac{\Gamma_m}{2\hbar}\right)}{\frac{(E-E_m)^2}{\hbar^2}+\frac{\Gamma_m^2}{(2\hbar)^2}}\right]$$

which is reduced to:

$$|f(E)|^2=\frac{1}{\pi}\sum_{m=1}^{N}\mathrm{X}_m^{\mathbb{R}}\frac{\frac{\Gamma_m}{2}}{(E-E_m)^2+\left(\frac{\Gamma_m}{2}\right)^2}+\frac{1}{\pi}\sum_{m=1}^{N}\mathrm{X}_m^{\Im}\frac{(E-E_m)}{(E-E_m)^2+\left(\frac{\Gamma_m}{2}\right)^2}$$

Supplementary Material References

(1) Herrera-Gomez, A.; Guzman-Bucio, D.; Cabrera-German, D.; Dutoi, A.; Vazquez-Lepe, M. O.; Cortazar-Martinez, O.; Carmona-Carmona, A.-J. Characterizing the Intrinsic Background in XPS Using the Narrow-Shirley Approach. *The Journal of Physical Chemistry C (submitted)* **2025**.

(2) Tougaard, S.; Sigmund, P. Influence of Elastic and Inelastic Scattering on Energy Spectra of Electrons Emitted from Solids. *Phys Rev B* **1982**, *25* (7), 4452–4466. https://doi.org/10.1103/PhysRevB.25.4452.

(3) Moeini, B.; Linford, M. R.; Fairley, N.; Barlow, A.; Cumpson, P.; Morgan, D.; Fernandez, V.; Baltrusaitis, J. Definition of a New (Doniach-Sunjic-Shirley) Peak Shape for Fitting Asymmetric Signals Applied to Reduced Graphene Oxide/Graphene Oxide XPS Spectra. *Surface and Interface Analysis* **2022**, *54* (1), 67–77. https://doi.org/https://doi.org/10.1002/sia.7021.

(4) Biesinger, M. C. Accessing the Robustness of Adventitious Carbon for Charge Referencing (Correction) Purposes in XPS Analysis: Insights from a Multi-User Facility Data Review. *Appl Surf Sci* **2022**, *597*, 153681. https://doi.org/https://doi.org/10.1016/j.apsusc.2022.153681.

(5) Morgan, D. J. Comments on the XPS Analysis of Carbon Materials. *C (Basel)* **2021**, *7* (3). https://doi.org/10.3390/c7030051.
(6) Gengenbach, T. R.; Major, G. H.; Linford, M. R.; Easton, C. D. Practical Guides for X-Ray Photoelectron Spectroscopy (XPS): Interpreting the Carbon 1s Spectrum. *Journal of Vacuum Science & Technology A* **2021**, *39* (1), 013204. https://doi.org/10.1116/6.0000682.
(7) Zborowski, C.; Renault, O.; Torres, A.; Yamashita, Y.; Grenet, G.; Tougaard, S. Determination of the Input Parameters for Inelastic Background Analysis Combined with HAXPES Using a Reference Sample. *Appl Surf Sci* **2018**, *432*, 60–70. https://doi.org/https://doi.org/10.1016/j.apsusc.2017.06.081.
